# Cellular and transcriptomic analyses reveal two-staged chloroplast biogenesis underpinning photosynthesis build-up in the wheat leaf

**DOI:** 10.1186/s13059-021-02366-3

**Published:** 2021-05-11

**Authors:** Naresh Loudya, Priyanka Mishra, Kotaro Takahagi, Yukiko Uehara-Yamaguchi, Komaki Inoue, Laszlo Bogre, Keiichi Mochida, Enrique López-Juez

**Affiliations:** 1 Department of Biological Sciences, Royal Holloway University of London, Egham, UK; 2 RIKEN Center for Sustainable Resource Science, Tsurumi-ku, Yokohama, Japan; 3 Kihara Institute for Biological Research, Yokohama City University, Totsuka-ku, Yokohama, Japan; 4 RIKEN Baton Zone Program, Tsurumi-ku, Yokohama, Japan; 5 Institute of Plant Science and Resources, Okayama University, Kurashiki, Japan

**Keywords:** Wheat, Plastid, Chloroplast, Leaf development

## Abstract

**Background:**

The developmental gradient in monocot leaves has been exploited to uncover leaf developmental gene expression programs and chloroplast biogenesis processes. However, the relationship between the two is barely understood, which limits the value of transcriptome data to understand the process of chloroplast development.

**Results:**

Taking advantage of the developmental gradient in the bread wheat leaf, we provide a simultaneous quantitative analysis for the development of mesophyll cells and of chloroplasts as a cellular compartment. This allows us to generate the first biologically-informed gene expression map of this leaf, with the entire developmental gradient from meristematic to fully differentiated cells captured. We show that the first phase of plastid development begins with organelle proliferation, which extends well beyond cell proliferation, and continues with the establishment and then the build-up of the plastid genetic machinery. The second phase is marked by the development of photosynthetic chloroplasts which occupy the available cellular space. Using a network reconstruction algorithm, we predict that known chloroplast gene expression regulators are differentially involved across those developmental stages.

**Conclusions:**

Our analysis generates both the first wheat leaf transcriptional map and one of the most comprehensive descriptions to date of the developmental history of chloroplasts in higher plants. It reveals functionally distinct plastid and chloroplast development stages, identifies processes occurring in each of them, and highlights our very limited knowledge of the earliest drivers of plastid biogenesis, while providing a basis for their future identification.

**Supplementary Information:**

The online version contains supplementary material available at 10.1186/s13059-021-02366-3.

## Background

Rational engineering of photosynthetic performance could provide the best available avenue to increase crop yield potential [1]. For such an approach to be undertaken, a fundamental understanding of chloroplast development is an absolute prerequisite. Leaves are the primary photosynthetic organs, within which mesophyll cells differentiate to become chloroplast-filled. Differentiation involves morphogenesis but also cell-appropriate organelle biogenesis programs. Understanding the build-up of photosynthetic capacity requires detailed knowledge of how these cells, and chloroplasts within, are produced and develop. Monocot leaves, where cell proliferation and differentiation are displayed along a linear developmental gradient, provide an ideally suited experimental system to study these processes. Leaf primordia form at the flanks of the stem cell population in the shoot apical meristem. Primordium cells, in contrast to stem cells, are already specified, have entered a period of maximal proliferation, but for a limited number of times, and can thus be considered the plant equivalent of progenitor cells in animal organs. Differentiation subsequently occurs in leaf primordia basipetally, resulting in a gradient of easily distinguishable cellular morphologies of distal differentiated cells towards the tip of the leaf, basal progenitor proliferating cells adjacent to the shoot apical meristem, and all possible intermediate stages in between.

This developmental gradient is common to the world’s three main cereal crops, wheat, rice, and maize. It is not dissimilar to the gradient of proliferation and differentiation along developing roots [2] but, in contrast, it provides a unique opportunity to study chloroplast biogenesis and differentiation. Pioneering early work made use of the developing maize or wheat leaf gradient to demonstrate that dumb-bell shaped, dividing chloroplasts appear at the base of the leaf [3, 4] and that chloroplast DNA replication occurs for longer than nuclear DNA replication does [5]. Later, detailed observations in developing barley leaves observed a very early accumulation of plastid DNA, with replication continuing in order to maintain genome content as plastids gradually proliferated, and with plastid transcription becoming established first for genetic machinery or, later, for photosynthetic genes [6, 7].

Chloroplast biogenesis involves a multiplicity of processes. These include the proliferation of a small number of proplastids, their preparation to synthesize large quantities of photosynthetic polypeptides encoded in the plastid genome, which itself requires replication of sufficient copies of the genome, activation of this endogenous genetic machinery and accumulation of ribosomes and other translation factors, development of the capacity for nuclear-encoded protein import into chloroplasts, synthesis of thylakoid galactolipid membrane and photosynthetic pigments, and synthesis or import and assembly of photosynthetic complexes in thylakoid membranes [8].

With the advent of whole genome information, this leaf developmental gradient has been exploited through a range of “omics” technologies. The maize leaf has received the most detailed attention, using transcriptomic, chloroplast “translatomic,” proteomic, phosphoproteomic, and metabolomic techniques [9–13]. Some of these studies also recorded structural features, such as the appearance of thylakoid membranes, and that of other organelles at different developmental stages [11, 12], but such record was descriptive and lacked quantitative data for cellular parameters that could be correlated with quantitative molecular events, to attain a comprehensive developmental map for mesophyll cell differentiation. Wheat, particularly hexaploid bread wheat with its large cell sizes, has proven particularly amenable to quantitative organellar analyses [14–16]. However, in wheat, given the recent genome decoding, transcriptome analysis is only available for whole leaf sheaths and blades [17]. In this work, we set out to capture the entire developmental gradient from meristematic to fully differentiated cells and to quantitatively understand chloroplast biogenesis, together with their underlying molecular processes, in order to describe a developmental trajectory of chloroplasts.

## Results

### Developmental analysis of the wheat leaf reveals stages of cell and chloroplast differentiation

In order to generate a quantitative analysis of chloroplast biogenesis and a simultaneous global gene expression map of the developing wheat leaf, we first carried out a careful selection of biological material. In preliminary experiments on consecutive leaves, we observed, as anticipated, rapid changes in cellular morphology across short physical distances at the base, and very limited differences at more mature stages. It is important to note that while the distance of cells from the leaf base is related to developmental time, the relationship is far from linear. Elegant measurements by Boffey et al. [15], of the first leaf of wheat grown under conditions similar to ours, identified the relationship between distance from the leaf base and cellular age after exit from the shoot meristem. We used this position/age relationship, corrected for the elongation rate observed in our conditions (see “Methods”), to estimate cellular age. The mesophyll cell morphology and the calculated cellular age prompted the need for much denser sampling at the base of leaves than towards the tip.

**Fig. 1:**
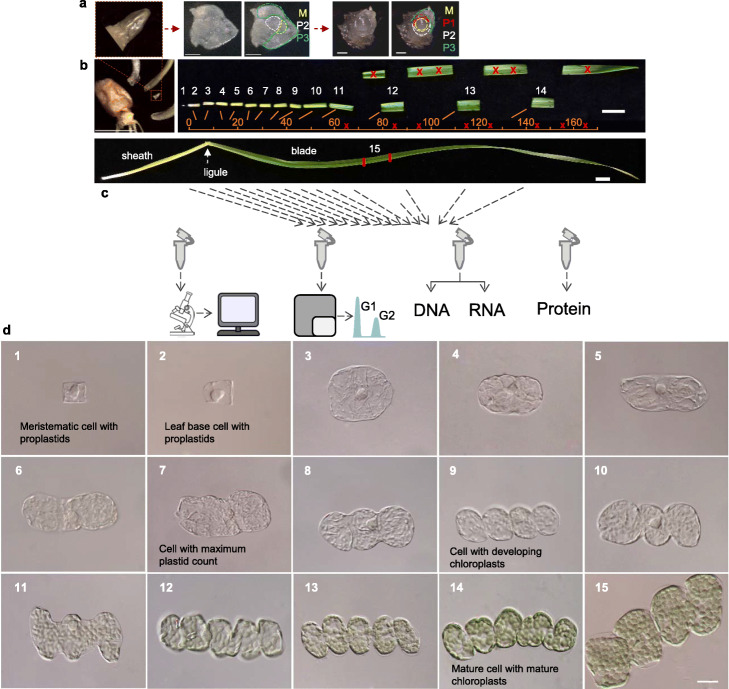
Developmental analysis reveals stages of cell and chloroplast differentiation. **a**, **b** Image of leaf sampling, showing the biological material used as sample 1 (**a**, shoot apex, 6-day-old) and 2–14, from 6-day-old, and 15, from 14-day-old leaf 1 (**b**). Scale bars: 100 μm (**a**), 5 mm for seed image and 10 mm for leaf dissection image (**b**). Cross marks indicate excluded sections. M: meristem. P1-P3: plastochron stages 1-3. **c** Experimental strategy. Parallel replicates were used for quantitative differential interference contrast microscopy, flow cytometric analysis of cell cycle, simultaneous DNA and RNA isolation and protein extraction. **d** Individual cellular morphologies of mesophyll cells, representative of each sample, visualizing cellular expansion following proliferation, increasing plastid number, acquisition of lobed cell morphology and filling of the available cellular space by expanding, green chloroplasts. Scale bar: 25 μm

Cereal leaves present two regions, the cylindrical basal sheath, which emerges last from the meristem and envelops younger leaves to provide structural support, and the blade, with photosynthetic role. A ligule separates them (see mature leaf in Fig. 1b). Sheath and blade cells inevitably undertake distinct developmental paths [17], and therefore it was important to select a leaf developmental stage before they become distinct, which under our conditions was the case for the first leaf of 6-day-old seedlings, which exhibited an essentially uninterrupted developmental sequence. In order to include the earliest fully proliferating cells, we also collected a sample of the shoot apical meristem with the incipient youngest leaves (plastochron stages P3 to P1), the primordium of leaf 3 being around 1.5 mm in length (Fig. 1a). The meristem produced only leaves, internodes initiating much later in development. To obtain a fully mature photosynthetic stage, but without any signs of senescence, we dissected the middle region of the 2-week-old leaf 1 blade to complete a total of 15 samples (Fig. 1b). We used the same dissected leaf samples to simultaneously obtain materials for quantitative microscopy-based cellular and organelle differentiation analysis, and cell cycle stage identification by flow cytometry and molecular analyses (Fig. 1c).

Our cellular analysis was focused on the photosynthetic mesophyll cells that make up about two thirds of the area of a transverse, mature C3 grass leaf section [18], and therefore of the leaf volume. Meristematic/leaf primordia cells were homogeneously small, prismatic, generally isodiametric and with a central large nucleus (Fig. 1d, sample 1). Cells at the leaf base (sample 2, first 5 mm) remained isodiametric, but increased in size. In the subsequent stage (sample 3, 5–10 mm), cells further enlarged, but remained isodiametric, while after the 10 mm position (samples 4–5) cells begun to elongate and after 15 mm started to produce the characteristic lobed shape of wheat mesophyll cells (sample 5 onwards). A large number of proplastids could be observed by varying the focal plane under DIC microscopy already in cells at early stages of leaf development, but greening, indicative of an assembled photosynthetic apparatus, became apparent only at around 30 mm from the meristem (sample 8 onwards), a few millimeters before the point at which leaf 1 emerged from its enveloping coleoptile. Thereafter, green chloroplasts grew rapidly in size and filled the available cellular space, arranged as a single layer sandwiched in a thin cytoplasm sheath between the vacuole and the plasma membrane (Fig. 1d).

Having identified the full range of cellular and organelle differentiation morphologies, representing the entire leaf developmental sequence, we proceeded to quantify the cellular and organellar parameters and the underlying molecular processes through global transcriptome profiling in the same 15 samples.

### Biologically informed gene expression map of wheat leaf development demonstrates key differentiation processes

**Fig. 2:**
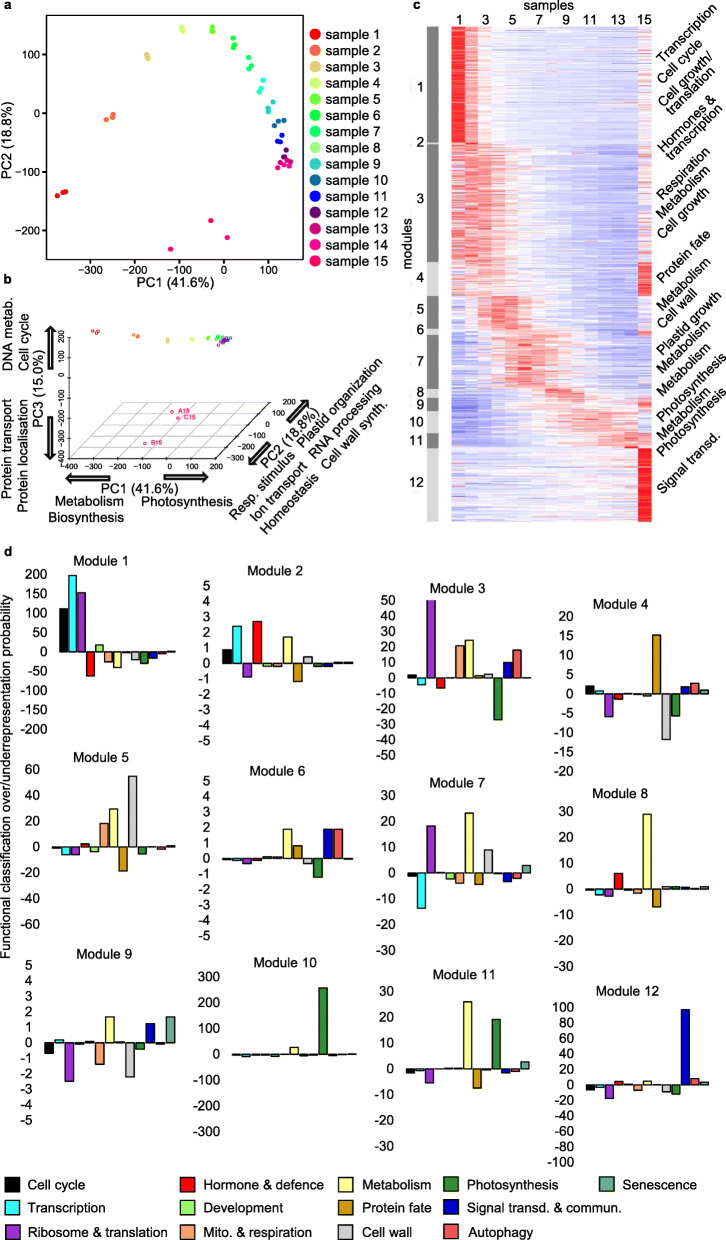
Biologically informed global gene expression program of the first leaf of wheat. **a** Principal component (PC) analysis (PCA), displaying the first two components of variance among each biological replica of each sample. **b** PCA displaying the first three components. Samples colored as in **a**. Ontology terms associated with the genes with the highest load in each direction of each PC are indicated in the corresponding axis. **c** Heatmap (red, high; blue, low) of the expression (*Z*-score) of 42,057 dynamically expressed genes (DYGs), grouped in clusters (modules 1–12) as identified by WGCNA, and ordered by timing of peak expression. Some selected, overrepresented functional classification terms (displayed in **d**) are included. **d** Probability (displayed as the inverse of the log_10_) of over/underrepresented functional classification terms of each gene cluster displayed in **c**

Triplicate RNA samples were subjected to reverse transcription and Illumina-based sequencing. Around 30 million reads were obtained per sample. We made use of the most recent IWGSC genome annotation [19], which encompasses close to 100,000 genes, including homoeologs of the A, B, and D genomes. We used principal component analysis of variance (PCA) to, in an unbiased manner, establish the degree of difference between the different samples and their replicas. A plot of first two principal components (*x* and *y* axes, Fig. 2a) or including the third one (*x*, *y*, and *z* axes, Fig. 2b) demonstrated a very short distance between the replica samples and therefore a high degree of reproducibility of the data. PCA also revealed that a broad coverage of the trajectory of the developmental gradient had been achieved, with the largest variance being observed during the earliest stages of cellular development. For example, while greening in sample 14 is over 30 fold greater than in sample 4 (Fig. 1b and see below), the variance between those two samples captured by both the first and second principal components (their distance along those two axes) is not as large as that between samples 1 and 4 (shoot apex and first 15 mm of the leaf base). The first three components accounted for nearly 80% of the total expression variance (Fig. 2c, d). In order to understand the biological processes represented by the principal components, we calculated the load factors of each gene for each of the three components, and identified gene ontology terms enriched in the genes with the top and bottom 5% load factors (see “Methods”). The result (Additional file 1: Figure S1) is summarized in the axes of Fig. 2b and shows that the first component, accounting for over 40% of the variance, effectively represented developmental (pseudo-) time, a gradual shift from early biosynthetic metabolism to photosynthesis. Interestingly, component 2 (19% of variance) moved forward and back, displaying an intermediate peak which, according to gene load factors, represents both plastid and cell wall organization genes that show maximum expression at samples 4 and 5 (the second cm from the leaf base). The third component involved a departure from DNA synthesis and an eventual peak of expression of mature tissue gene signatures (transport processes).

Following a selection procedure designed to compare the different points across a temporal sequence, with thresholds for minimum expression, fold change, and coefficient of variation (see “Methods”), a total of 42,057 dynamically expressed genes [20] (DYGs, including individual homoeologs) were identified (Additional file 2: Table S1). This constitutes over 40% of the entire genome. Clustering using WGCNA [21] identified 12 expression modules (Fig. 2c), with peaks of expression covering the full developmental gradient. We used an over- or under-representation procedure of gene sets representing selected biological and molecular functions (Fig. 2d), which allowed both a broad representation of the processes and the ability to visualize sub-modules within the same functional groups (Additional file 1: Figure S2) [22]. The first module, with peak of expression in the shoot apex, is enriched to an extraordinary degree (*p* < 10^−100^) in genes involved in transcriptional control, cell cycle, and translation (including genes encoding ribosomal proteins), i.e., cytoplasmic cell growth. The second module was small but highly specific to the leaf base and is almost-uniquely overrepresented in hormone-related functions. The third module showed a broad peak in the early samples and encompasses further ribosomal and mitochondrial build-up. These three modules alone included nearly half (47%) of all DYGs.

Examination of the expression of genes selected as part of the functional classification strategy (Additional file 1: Figure S2, Additional file 3: Table S2) provided further, highly informative insight into the processes of construction of the leaf organ. Given that the processes of “transcription and its regulation,” “translation,” and “protein fate” in particular could represent markedly different activities in different cellular compartments, we further indicated through color codes for these three processes whether the individual genes encoded proteins targeted to the mitochondrion, the chloroplast, both or elsewhere (Additional file 1: Figure S2, Additional file 3: Table S2). We observed that while the majority of genes for ribosomal or translation-related proteins were tightly co-expressed and highly active at the shoot meristem and the first leaf base samples, and this was also the case for mitochondrial translation proteins, genes encoding chloroplast ribosomal and other translation-related proteins were particularly abundant among the cohorts of genes peaking in samples 4–11, 10–60 mm from the base. Build-up of the mitochondrial metabolism and respiratory chain peaked in samples 3–4, between 5 and 15 mm, while the majority of photosynthesis-related genes became substantially expressed from sample 8, after 30 mm from the base. Tight cohorts of genes within metabolism or cell wall synthesis and modification also reveal discrete and sequential biogenic activities (Additional file 1: Figure S2, Additional file 3: Table S2). We noted that the unique gene expression signature of the mature leaf sample was not primarily due to the initiation of senescence or of autophagy, as the overrepresentation of specifically these two processes in it was limited, genes involved in many other functions also being altered.

The function of the shoot apical meristem, cell specification processes in leaf primordia, and later differentiation, of both cells and chloroplasts, involve fundamental regulatory events brought about by hormonal action [23, 24]. Genes functionally classified as of hormone action were overrepresented specifically at the leaf base, sample 2 (Fig. 2c, d). We took advantage of our transcriptome data to indirectly examine the broad extent of action of eight plant hormones, visualizing the expression of genes involved in their synthesis/catabolism or signal transduction, and that of genes previously shown to be induced by the relevant hormone (serving as reporters), through a previously used approach [24, 25]. Particularly important roles for auxin were evident in the shoot meristematic region and the base of the leaf (Additional file 1: Figure S3, Additional file 4: Table S3). Auxin action occurs through strikingly distinct cohorts of genes in the shoot apical region, the leaf base, and the regions in which different stages of cell elongation occur (Additional file 1: Figure S3). The data also suggest differential gene functions for auxin receptors, expressed in the shoot apex containing meristematic cells (TIR1), in specified progenitor cells at the leaf base (AFB5) or in early expanding cells (ABP1) (Additional file 4: Table S3). The same applies to various auxin response factors (ARFs), showing sequential expression with peaks ranging from the shoot apex to the region of cell expansion (MONOPTEROUS/ARF6/ARF8/ARF19). In relation to chloroplast differentiation, this approach highlighted a possible role only for cytokinin, given that about half the cytokinin signalling genes displayed showed clearest expression between samples 6 and 13 (Additional file 1: Figure S3) at the time during which greening was most pronounced. These signatures generate a wealth of hypotheses, concerning leaf and organelle development, for further analysis.

### Consecutive occurrence of cellular proliferation, cytoplasmic growth, and cell expansion

**Fig. 3:**
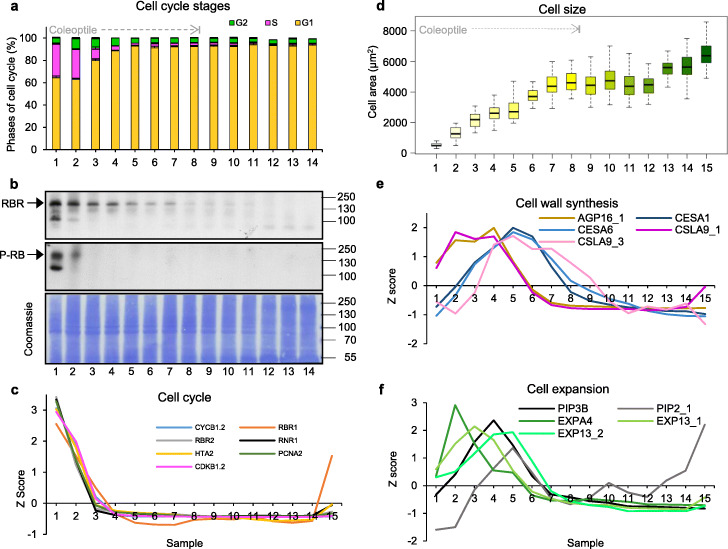
Very early cell proliferation and subsequent cell growth coincide with expression of associated genes. **a** Summary of flow cytometric analysis of cell cycle stages for the first 14 samples. G1: gap 1. G2: gap 2. S: DNA synthesis phase. **b** Immunoblot of 20 μg of total protein from each of the first 14 samples, using antibodies specific to retinoblastoma-related protein 1 (RBR1) and its phosphorylated form (P-RB). The lower panel shows a replica gel stained for total protein with Coomassie brilliant blue. Molecular weights (kDa) are indicated on the right. **c** Expression of cell cycle-associated genes, displayed as *Z*-score. The individual genes are listed in Additional file 5: Table S4. **d** Box plots of cell size, measured as cell plan area, for each sample. See Additional file 5: Table S6 and Table S7 for details of microscopy calculations (*n* = 48 cells per sample) and for R script. The boxes represent upper and lower quartiles, with the middle line the median, and the whiskers the full range of observations. The fact that the leaf material for the first 8 samples was included within the seedling coleoptile is indicated. **e** Expression (*Z*-score) of cell wall synthesis-associated genes. **f** Expression (*Z*-score) of cell expansion genes

Multiplication of organelles is essential for their number to be maintained in proliferating cells, when each cell division on average halves it, as well as to increase their number in non-dividing cells as part of cellular differentiation. Thus, the quantitative understanding of chloroplast biogenesis necessitates the simultaneous understanding of cell proliferation. We therefore examined this in detail, using quantitative microscopy (Fig. 1) and simultaneous flow cytometric analysis of cell cycle stages (Fig. 3a, Additional file 1: Figure S4). In sample 1, containing the meristem/leaf primordia, we found a high proportion (around 30%) of nuclei undergoing S phase. Based on data showing that the fastest recorded cell cycle in wheat is 12 h [26] while the duration of S phase is around 3 h [27], it is likely that the totality of meristem and primordia cells in our sample 1 are undergoing cycling and that the cycle is operating at full speed. The S phase proportion declined slightly in sample 2, the first 5 mm of the leaf base, very rapidly diminished to less than 30% of that in the shoot apex in sample 3, 5–10 mm from the leaf base, making the cells’ doubling time close to 2 days, and became barely detectable above background subsequently (Fig. 3a, Additional file 1: Figure S4).

We selected a number of signature genes representative of both DNA synthesis and mitosis (listed in Additional file 5: Table S4) from our transcriptome data, and represented their relative expression levels as *Z*-scores (the expression values for these and other genes in this functional class, as those in others, are provided in Additional file 3: Table S2). In agreement with the flow cytometry data, these genes peaked in expression in the shoot apex, and their transcript levels became minimal in sample 3, between 5 and 10 mm of the leaf base (Fig. 3c). These cell cycle genes are known targets of the E2F transcription factors, which are themselves known to become active when the RETINOBLASTOMA-RELATED (RBR) protein is inactivated by phosphorylation [28, 29]. Immunoblot analysis shows that RBR phosphorylation is high only in the meristematic sample and already declines substantially in the leaf base, becoming undetectable subsequently (Fig. 3b). The total level of RBR1 (one of two RBR proteins present in monocots, and against which an antibody is available) is also most abundant towards the leaf base, but diminishes less rapidly than its degree of phosphorylation does, consistent with its role in repression of cell cycle genes at those subsequent stages.

Measurements of cell size showed an increase already at the leaf base compared to cells in the shoot apex, indicating that the cell expansion program starts at the base while cells still proliferate (Fig. 3d). The vast majority of cell expansion occurred up to sample 7, 30 mm from the base, when cells are less than 2 days old; making this the region which drives leaf lengthening [15]. A small second bout of expansion of mesophyll cells (likely increase in width) occurred from sample 12, after 80 mm, accounting for 15–20% of the final cell plan area (Fig. 3d). Signature genes for cell wall synthesis (cellulose synthases, arabinogalactan proteins, Additional file 5: Table S4) showed two distinct early peaks (Fig. 3e), with the main cellulose synthases peaking around sample 6, 20–25 mm from the leaf base. Genes associated with cell expansion or turgor facilitation (expansins, aquaporins) showed a corresponding early expression (Fig. 3f).

In summary, divisions are rapid and continuous in leaf primordia. At the base of the developing leaf, cells only undergo between one and two further rounds of division. In the first half day after leaving the meristem, cells move up about 2–3 mm [15] by the expansion of dividing, preceding cells also leaving the meristem. Between half and 1 day, cells move up to 8–9 mm and essentially cease proliferation. Simultaneously with proliferation, cells initiate expansion at the leaf base, this continues after cells fully exit the division cycle, and largely concludes in the following 24 h, ending the first phase of morphological differentiation (Fig. 3d).

### A phase of plastid proliferation is followed by the build-up of plastid genomes and transcription and translation machinery

**Fig. 4:**
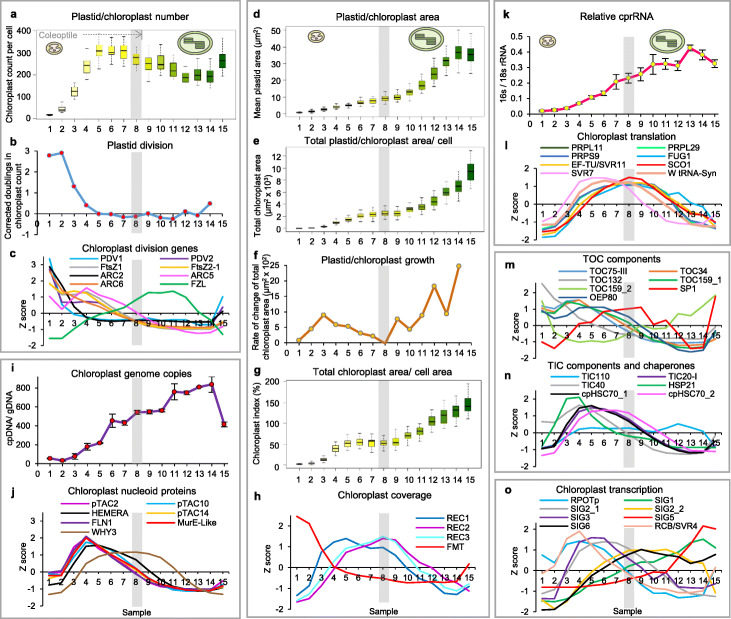
Distinct plastid and chloroplast growth phases, the plastid phase involving proliferation, genome replication and ribosome build-up. **a** Plastid number per cell. The region covered by coleoptile is shown. **b** Calculated plastid division rate. See Additional file 5: Table S6, S7 and S8 for details of microscopy calculations (*n* = 48 cells per sample) and cell proliferation corrections. **c** Expression (*Z*-score) of nuclear-encoded genes for plastid division-associated proteins. **d** Individual plastid/chloroplast size, measured for plastids positioned perpendicular to the direction of view (*n* = 10 plastids per cell). **e** Total plastid/chloroplast area per cell. **f** Chloroplast cellular growth rate, corrected for the effect of cell division, as in **b**. **g** Chloroplast index, ratio of the total chloroplast plan area in each cell and the plan area of the same cell. **h** As **c**, for proteins impacting the cellular chloroplast coverage. **i** Ratio of chloroplast genome (cpDNA) to haploid nuclear genome (gDNA) copies, measured in 3 biological replicates, each measured as 2 technical replicates, with the mean displayed. Error bars represent standard error of the mean between biological replicates. **j** As **c**, for plastid transcriptionally active chromosome proteins. **k** Ratio of chloroplast ribosomal RNA (cprRNA) to cytosolic ribosomal RNA (rRNA), quantified in each of four independent RNA preparations for each of the 15 samples. Values displayed as means ± standard error of the mean. **l** Expression, as **c**, for chloroplast translation-related proteins. **m**, **n** As **c**, for chloroplast protein import-associated translocon components. **o** Expression of genes for chloroplast transcription-related proteins. All individual genes are listed in Additional file 5: Table S4. The region of transition between the plastid and chloroplast growth phases, around sample 8 (identified in Fig. 4f), is shown as a gray band in every panel

The most important aspect of mesophyll cell differentiation, and arguably of leaf function, is the gradual filling of cells with plastids (Figs. 1d, 4). We used quantitative microscopy [30] to record the number of plastids or chloroplasts, their size and proportion of the cell they occupied, and used quantitative PCR and alternative techniques to measure the number of copies of the plastid genome and of plastid ribosomes in relation to their cytosolic counterparts (Fig. 4).

Plastid number per cell increased in cells located within the basal 20 mm, up to sample 5 (Fig. 4a). Considering that in the first two samples cells halve the plastid number at each division, the calculated frequency of plastid divisions was in fact highest in proliferating cells but declined slower than cell proliferation did (Fig. 4b). Those data show that once cells become specified at the base of the leaf, their plastids undergo between 4 and 5 rounds of division in total. This is confined largely to the first 24 h and fully to the basal 15 mm segment, samples 2–4, broadly coinciding with cell enlargement but before the lobed cell morphology is attained. A selection of plastid division signature genes (Additional file 5: Table S4) was consistent with such a pattern, the expression of several plastid division genes is early and mirrors the calculated division, see, e.g., *ARC2*, ceasing after 15 mm, although others extend further (Fig. 4c). The *FZL* gene has been shown to be involved in thylakoid biogenesis [31], but its mutant phenotype [32], with fewer, larger chloroplasts, is suggestive of a possible role in plastid proliferation; it is interesting to observe its later expression, between samples 6 and 12, 20 to 80 mm from the base (Fig. 4c), compatible with thylakoid development and incompatible with a role at the stage of division. To our surprise, we consistently observed a small, gradual reduction of plastid numbers in the latter region. The reason for both of these observations will require future investigation.

The size of individual plastids increased continuously from the very early stages, even during plastid proliferation, but reached a transient plateau before greening and then underwent a second phase of rapid enlargement (Fig. 4d). Remarkably, the total area of all plastids in a cell also increased in two distinct stages (Fig. 4e). The rate of growth, corrected for the effect of cell division, even more clearly showed the two distinct phases of build-up of the chloroplast compartment. The first, which we designated “plastid” phase, preceded greening and essentially concluded with the stage in sample 7, at 30 mm from the base (between 1.5 and 2 days after cells left the meristem), while the second, which we consider “chloroplast” phase, began at around sample 10, 40 mm from the leaf base and continued throughout to fill the mesophyll cells (Fig. 4f). This is intriguing since it reveals a spurt of plastid growth activity well before greening. The transition between the two phases broadly coincides with the emergence of the first leaf from its enveloping coleoptile, a translucent, non-photosynthetic, leaf-like structure, which aids leaf emergence through the soil and provides structural support. The proportion of the cell occupied by the organelles, i.e., the cellular chloroplast compartment or “chloroplast index,” was calculated as the total area of chloroplasts (obtained as the product of chloroplast number and average chloroplast plan area), divided by total cell plan area. We found this also to follow a clear biphasic profile (Fig. 4g). The full occupancy of cells by chloroplasts is dependent on the activity of the *REC* gene family [33]. We found (Fig. 4h) that while *FMT* showed expression consistent with a role in mitochondrial cellular distribution, *REC1* peaked around the time of maximal plastid growth, while the expression of *REC2* and *REC3* preceded the “chloroplast growth” phase.

Concomitant and subsequent to their multiplication, starting from a small initial number of proplastids, the replication of sufficient copies of the plastid genome follows, to support the synthesis of large quantities of the photosynthetic polypeptides it encodes. Indeed, DAPI staining of DNA showed that while the majority of cellular DNA is nuclear, non-nuclear DNA in mature mesophyll cells was associated with chloroplasts (Additional file 1: Figure S5). In agreement, we observed (Fig. 4i) that the detectable but very low initial number of copies of chloroplast DNA (cpDNA) per haploid nuclear genome (gDNA) underwent multiple rounds of replication throughout the “plastid growth” phase, with less than one final round (not all copies of cpDNA replicated) taking place during the “chloroplast growth” stage. Multimers of cpDNA and associated proteins form nucleoids. A number of such polypeptides have been identified as plastid transcriptionally active chromosome proteins (pTACs) [34]. The expression of several of these nucleoid proteins also largely peaked during the plastid phase (Fig. 4j), although that of the wheat homolog of pTAC12/HEMERA (see later) continued for longer and that of pTAC11/WHIRLY3 followed a distinct profile, more akin to that of chloroplast translation-associated proteins (see below). We also observed an apparent final loss of around 50% of the cpDNA in the mature leaf sample. Given the fact that we quantified three different plastid DNA genes, in the so-called large and small single-copy regions and in the inverted repeat (Additional file 1: Figure S6), this decrease cannot be explained by plastid genome rearrangements. The decrease, however, is consistent with a smaller decrease observed in mature maize leaf stages in one study [35], and does not support the near-total loss observed in another study [36].

While the vast majority of plastid-encoded proteins play a photosynthetic role, chloroplast ribosomes are constituted of plastid-encoded rRNA, making an early, active plastid genome essential. Like chloroplast genomes, chloroplast ribosomes, as quantified by the content of 16S cprRNA relative to 18S cytosolic rRNA, were present in very low amounts in shoot apical or leaf base cells, and accumulated largely during the plastid growth phase, by that time achieving already more than 50% of their final content in spite of the small total plastid content (Fig. 4k, Additional file 1: Figure S7). This was corroborated using two separate techniques (see “Methods”). As a result, the investment of cellular translation capacity clearly shifts from almost entirely cytoplasmic (less than 1% plastidic), when cell proliferation is taking place, to more balanced (between 1/5 and 1/3 of total rRNA being plastidial), for almost the entire duration of the greening process. Genes for nuclear-encoded chloroplast proteins constituting part of ribosomes or otherwise associated with chloroplast translation exhibited the broadest profiles in expression, spanning both the plastid and the chloroplast phases (Fig. 4l), raising rapidly in sample 3, after the first 5 mm of leaf base (in cells of under 1 day of age since leaving the meristem) and remaining high until at least 80 mm, in sample 12 (2 days later).

To support chloroplast biogenesis, the capacity for the import of nuclear-encoded proteins into chloroplasts needs building up. Our data show that the early plastid phase coincides with peaks of expression of genes for several protein import translocon components, at the outer and inner plastid envelopes (samples 3–4, 5 to 15 mm from the meristem, Fig. 4m, n, Additional file 5: Table S4). These components include homologs of TOC34, TOC159 (I) and the channel TOC75. Meanwhile, an alternative TOC159 (II), expressed in cells at the shoot apex, reinitiated expression later. Of note, the gene for the SP1 ubiquitin ligase, involved in Arabidopsis in the remodelling of import complexes to switch from an import function for housekeeping to one for photosynthetic polypeptides, or vice versa [37], reached highest, broad levels of expression around the transition point from the plastid to the chloroplast phase (Fig. 4m).

Transcription occurs in plastids at nucleoids. In agreement with previous observations [38], different actors of plastid transcription were synthesized in succession, with the nuclear-encoded RNA polymerase (RPOTp, homologous to the mitochondrial polymerase) being expressed early (Fig. 4o). Our nuclear transcriptome data do not include the expression of the subunits of the multimeric, alternative, chloroplast-encoded RNA polymerase, also known as plastid-encoded polymerase or PEP. However, the SVR4/RCB (regulator of chloroplast biogenesis) protein has recently been shown to play a central role in PEP assembly [39], and its gene expression profile (Fig. 4o) matches those of both the RPOTp and that which had been followed by several pTACs (Fig. 4j) whose loss indeed impacts early PEP function [34]. In a very revealing manner, sigma factors, the nuclear-encoded regulatory subunits of the chloroplast-encoded polymerase, peaked in expression successively, in the order of SIG3, SIG2_1 (plastid stage), SIG2_2, SIG6 (transition) and SIG1, SIG5 (chloroplast stage), suggesting dedicated function in expression of different cohorts of PEP-dependent genes underpinning the phases of chloroplast biogenesis.

### The latter phase of chloroplast development involves photosynthetic build-up

**Fig. 5:**
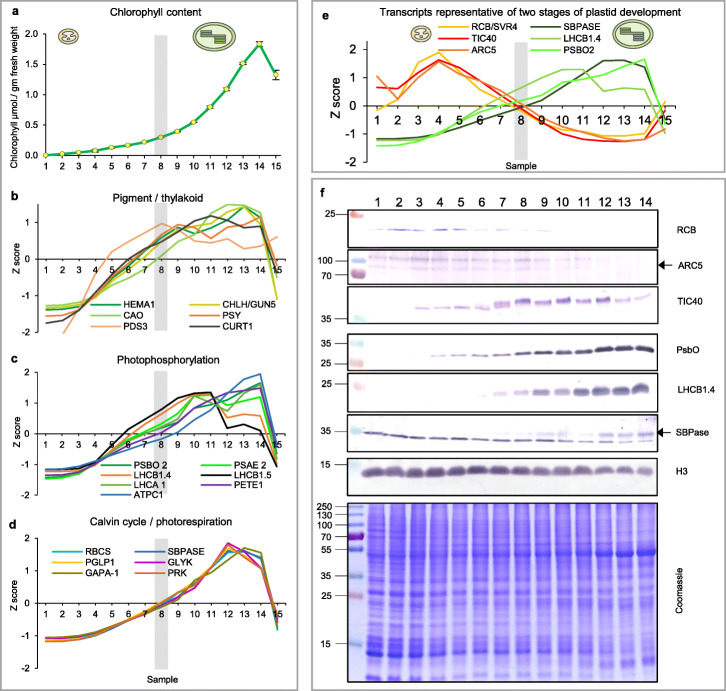
The second, chloroplast growth phase involves greening and is supported by protein accumulation profiles. **a** Chlorophyll content, quantified in each of three independent biological replicates per sample. Error bars represent standard error of the mean. See Additional file 5: Table S6 for calculations. **b**, **c**, **d** Expression (*Z*-scores) of pigment biosynthesis and thylakoid biogenesis (**b**), light reactions (**c**) and carbon fixation-associated genes (**d**). **e** Expression (*Z*-scores) of chloroplast development-associated transcripts reflecting two stages of plastid development, peaking in the early plastid phase (*RCB*, *ARC5* and *TIC40*) and, second, chloroplast phase (*PSBO2*, *LHCB1.4* and *SBPAse*). **f** Immunoblot analysis of the protein products of the genes displayed in **e**. In total, 20 μg of protein of samples 1–14 (for PSBO2, LHCB1.4 and SBPAse), 40 μg (for RCB, ARC5 and TIC40) or 10 μg (for Histone H3 as a constitutive control) was separated on denaturing SDS-PAGE gels, transferred to blots and probed with antibodies against the protein indicated. A Coomassie-stained total protein replica gel is also shown. Molecular weights (KDa) are indicated on the left. The results show one typical example from among three independent protein extraction and immunoblot experiments

We carried out a bulk quantitation of the development of photosynthetic apparatus (chlorophyll-containing reaction centers and antenna proteins) by measuring chlorophyll per unit leaf mass (Fig. 5a). This showed that pigment-containing complexes accumulate gradually but very slowly in young cells undergoing the plastid expansion phase, their rate of accumulation becoming substantial only around sample 7, 30 mm (cell age between 1.5 and 2 days), as chloroplasts initiate their rapid growth phase, starting from roughly 30% of their final individual area.

Signature genes (Additional file 5: Table S4) were used to characterize the development of the photosynthetic apparatus. Their relative expressions were remarkably consistent, but exhibited developmental (pseudo-) time shifts: genes involved in chlorophyll and carotenoid biosynthesis initiated their expression early, in sample 4, between 1 and 1.5 days of cell age, while plastids were still proliferating; the *CURT1* gene, whose product plays a structural role in thylakoid membrane development [40], followed an identical profile (Fig. 5b). Genes for nuclear-encoded proteins associated to the reaction centers, or for antenna or electron transport components followed similar kinetics (antenna transcript levels dropping earlier), but were shifted by a few hours (at this stage by about one sample, Fig. 5c). Carbon fixation-related genes followed almost immediately after (Fig. 5d). Notably, genes for photorespiration-related chloroplast enzymes followed essentially identical patterns to those for carbon fixation (Additional file 5: Table S4, Fig. 5d), consistent with shared regulation. In summary, the distinct plastid and chloroplast phases of organelle biogenesis are underpinned by corresponding, distinct gene expression programs to synthesize and assemble the photosynthetic capacity.

We then set out to determine how transcript levels are reflected in the abundance of plastid-localized proteins during the distinct stages of plastid biogenesis. To this end, we used immunoblots to monitor the levels of three protein products of genes representing each of the two phases. SVR4/RCB, ARC5 and TIC40 represent fundamental agents in PEP assembly, plastid division and protein import respectively. Proteins that are part of the photosystem antenna (LHCB1), are associated with the PSII reaction center (PsbO) or form part of the carbon fixation cycle (SBPase) were also selected. Transcripts for these genes accumulated in cells at the “plastid growth” (*RCB*, *ARC5*, *TIC40*) or “chloroplast” phases (*LHCB1*, *PsbO*, *SBPase*, Fig. 5e). Immunoblots demonstrated a clear plastid- (RCB, ARC5) or chloroplast-phase (LHCB1, PsbO, SBPase) protein accumulation profile, while TIC40 protein, in spite of being the product of a “plastid stage” transcript, was abundant through both stages, presumably a result of low protein turnover and of a role which is also fundamental for photosynthetic chloroplast differentiation (Fig. 5f). Interestingly, TIC40 appeared as two forms of slightly different electrophoretic mobility, the transition between them coinciding almost exactly with the plastid/chloroplast stage transition. The nature and significance of this transition is currently unknown. These protein data provide further support for the distinct phases of plastid biogenesis.

### Stage-specific modelled activity of candidate regulators of chloroplast biogenesis

In an effort to associate candidate drivers to the two phases of the chloroplast biogenesis gene expression program, we examined the expression of previously identified proteins with either chloroplast-related transcription factor function, or a range of other functions but which also impinge on the regulation of transcription for chloroplast proteins. Such regulators have been identified by mutants leading to defects in greening (G2/GLK [41]) or the response to light (HY5 [42], HEMERA/pTAC12 [43], RCB/SVR4/MRL7 [39] and NCP/MRL7-L [44]) or cytokinin (GNC [45]). Supporting evidence of the regulatory roles of GLKs and GNC is the fact that, when overexpressed, they promote ectopic greening of excised Arabidopsis roots [46]. CIA2 was identified through its involvement in the expression of chloroplast protein translocon components [47]. While not a direct transcriptional regulator, we also separately monitored expression of the *GUN1* gene, whose product is central for chloroplast-to-nucleus (retrograde) communication, which itself has a major impact on nuclear gene expression [48].

**Fig. 6:**
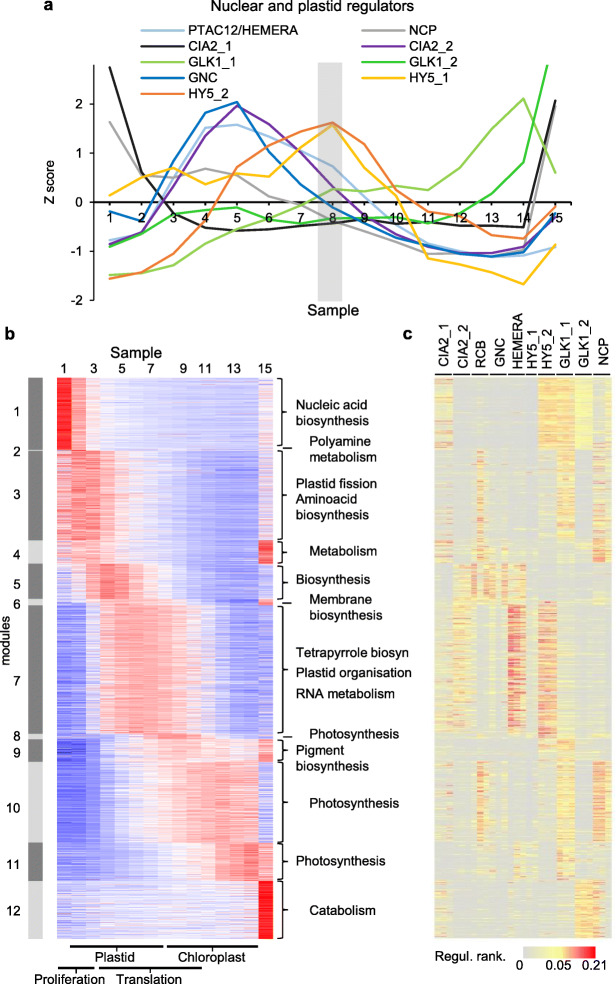
Gene regulatory network-predicted extent and limitation of known candidate regulators. **a** Expression (*Z*-score) of previously known candidate nuclear positive transcriptional regulators of plastid or chloroplast development. The region of transition between the plastid and chloroplast growth phases is shown as a gray band. The individual genes are listed in Additional file 5: Table S4. **b** Expression of all DYGs for chloroplast-targeted proteins (see “Methods”), displayed according to WGCNA modules as identified in Fig. 2c. Terms on the right show typical overrepresented gene ontology terms for the function of genes in the module. Bars at the bottom refer to the organelle development processes established in Figs. 4 and 5. **c** GENIE3-generated gene regulatory network providing best-estimate links (or their absence) between previously known chloroplast-related positive transcriptional regulators (**a**) and DYGs encoding chloroplast proteins (**b**); the color scale indicates regulatory ranking, gray indicating absolute lack of predicted regulatory association. The separate columns represent the independently computed values for the different homeologs (in most cases three) of each candidate regulator

*GNC*, *RCB*, *HEMERA* and one *CIA2* homolog were found to coincide in expression with the plastid expansion phase (Fig. 6a; for *RCB* see Fig. 5e). *HY5* was expressed early but peaked in expression at the plastid/chloroplast growth transition stage, which occurs approximately at the stage of leaf emergence from the translucent coleoptile into full light (Fig. 6a). Two *GLK1* homologs exhibited elevated expression during the chloroplast greening phase, consistent with their known targets, although *GLK1_2* also showed a degree of both early and very late expression, possibly suggestive of further plastid development and chloroplast resource mobilization roles. Only a second *CIA2* homolog (which we name *CIA2_2*) and *NCP* exhibited expression potentially associated with the very active plastid proliferation and growth phase (Fig. 6a).

Of interest, the central “retrograde” communication gene *GUN1* was maximally expressed at the transition stage between the plastid and chloroplast phases (Additional file 1: Figure S8). *GUN1* encodes a pentatricopeptide repeat (PPR) protein. Members of the PPR family are involved in RNA editing, turnover or other processes of metabolism of RNA for components of the translation machinery, as PPR5 or PPR103, or for individual subunits of photosynthetic complexes, as HCF152 or PPR10 [49] or for both kinds of proteins as PGR3 [50]. The profiles of these *PPR* genes reflected those functions, but that of *GUN1* at the transition stage was unique among them (Additional file 1: Figure S8), and this may have implications for our understanding of its biological role.

Light-dependent chloroplast development is also known to involve the inactivation of PIFs, transcription factors of the bHLH superfamily and negative regulators of chloroplast development in the dark, which are rapidly turned-over in the light [51, 52]. Homology searches to known monocot PIFs [53] identified five sets of wheat homoeologous genes, homologous to either *PIF1* or *PIF3*. Their expression (Additional file 1: Figure S9a) was largely restricted to samples 6 onwards (over 20 mm from the meristem), mostly during the chloroplast growth phase. The exception was *PIF3.2*, whose expression was limited to the plastid phase (Additional file 1: Figure S9a). Other indirect negative regulators of light responses are the *COP1* and *DET1* genes, COP1 protein being involved in the turnover of positive regulators like HY5 in the dark, or negative regulators like PIF1 in the light [53]. *DET1* exhibited a late plastid-phase expression, also the time of maximal plastid growth, while that of *COP1* was chloroplast-phase-specific (Additional file 1: Figure S9a).

We examined whether the candidate transcriptional regulators could be associated with the gene expression program underpinning chloroplast biogenesis. To this end, we sought a prediction of potential links between target genes (all genes for plastid-localized proteins) and their candidate regulators using a computational algorithm. We first assembled the target list by filtering the DYGs (Fig. 2c) for those encoding proteins with a predicted or previously observed plastid localization (Fig. 6b, Additional file 1: Figure S9b, Additional file 6: Table S9, see “Methods”). We then used GENIE3, a top-performing gene regulatory network reconstruction tool employing a random forests algorithm [54] to select the most likely candidate regulator from among the above known regulatory genes. We did this together for all candidate regulators, but visualized the result separately for positive and negative ones, as heatmaps of rankings of association calculated by GENIE3, rather than as a classic network. This was because the heatmap display better reflects both possible outcomes, presence or absence of connectivity between target and regulator. Figure 6c represents for candidate positive regulators the result, in which the color reflects the likelihood of regulation of a gene for a chloroplast protein in the corresponding row in Fig. 6b, by the regulator in the corresponding column in Fig. 6c. This result (see also Additional file 7: Table S10) predicted substantial roles for RCB and also (unexpectedly) GLK1_1 homologs during the plastid build-up stage, for HEMERA to the large group of genes which includes, among others, those for plastid ribosomal proteins, and again for GLK1_1 during the chloroplast build-up stage. It also showed very limited connectivity for the only candidates with early expression, homologs of CIA2 and NCP, or for any other candidate regulators, to genes active during the early stages of plastid build-up, for example when proliferation occurs (Fig. 6c). As for potential negative regulators (Additional file 1: Figure S9c), if considered taking into account the fact that the regulatory role would be repressive, the result pointed to only a low ranking role for PIF3.2 in the plastid build-up phase.

## Discussion

The analysis of the developing cereal leaf has been a powerful approach to reveal early events in cellular and organelle differentiation [10–13, 55]. Our study has generated the first detailed gene expression map to date of the developing leaf of wheat, one of the world’s most important three crops. It has also produced a dataset of (1) unprecedented resolution, when compared to previous monocot leaf gene expression analyses, and (2) unique content, because of its combined transcriptomic and quantitative cellular analyses. This approach has allowed us to observe from the earliest stages of mesophyll cell differentiation up until the fully mature stages as a continuum. Simultaneously, we characterized the evolution of the chloroplast compartment from proplastids in meristematic cells until fully developed chloroplasts. Capturing early stages of leaf development has been instrumental to uncover the complexity of the plastid phase before greening, and distinguish this from the subsequent, more easily observable second phase of green chloroplast differentiation.

While a notion of two distinct stages of chloroplast development has been put forward previously [56], this referred to greening of an Arabidopsis single-cell culture upon transfer to light, which resulted in two waves of photosynthetic gene expression. The two phases identified in our leaf developmental study are clearly distinct, encompassing an early plastid multiplication and establishment phase, prior to the second chloroplast build-up stage which involves photosynthesis-related genes. A very recent, and elegant, structural and biochemical analysis of greening chloroplasts of Arabidopsis cotyledons in the light has also observed two distinct phases [57]. However, these involved a plastid “structure establishment” stage followed by a chloroplast greening one, during which the bulk of organelle proliferation also occurred. Therefore, the greening of previously dark-grown, etioplast-filled but unexpanded cotyledon cells involves a somewhat distinct sequence of processes to that we observe here, with the processes we observed in the developing wheat leaf arguably being more representative of the bulk of photosynthetic differentiation in nature. Based on gene expression signatures, elements of the first phase of plastid development have been observed, by a study of consecutive emerging leaves, in plastochron stage 4 of very young rice plants [58]. Our data demonstrate the very early occurrence of plastid proliferation, at the beginning of the plastid phase, and they further show that build-up of the chloroplast translation machinery spans the plastid and chloroplast stages. The key significance of our study is the comprehensive identification of processes contributing to the build-up of the chloroplast compartment, to facilitate linking these to known—and enable to search for novel—regulators.

### Events in cellular and organellar life history

**Fig. 7:**
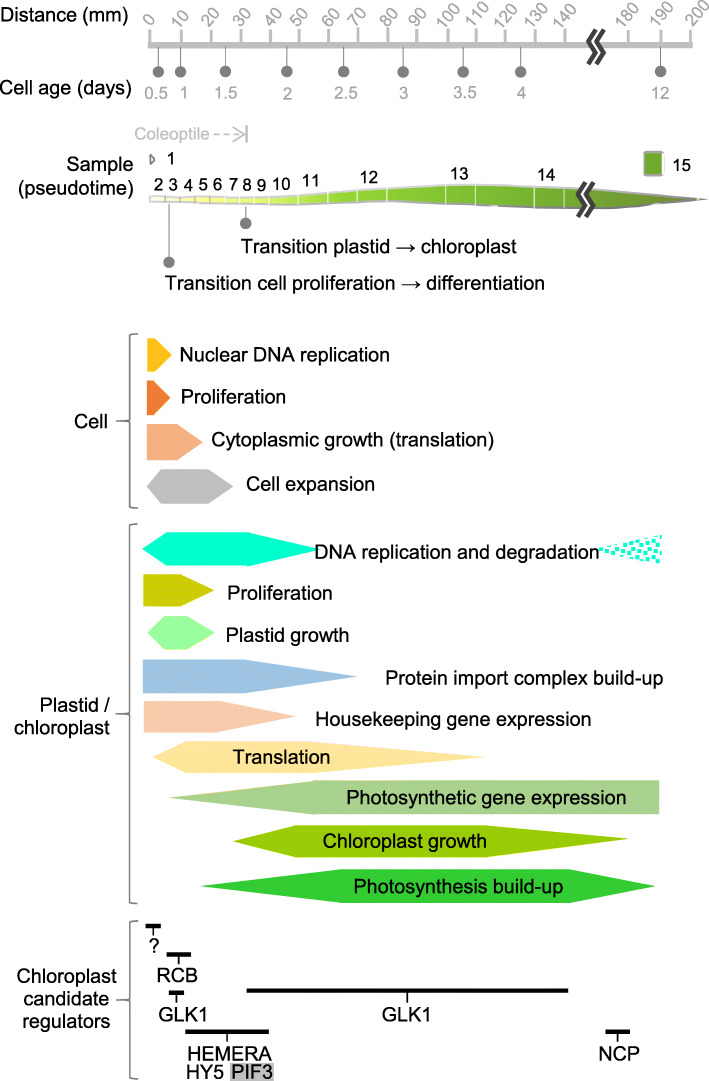
Summary of process underlying cellular and plastid or chloroplast differentiation. Summary of elementary biological processes involved in cell (top) and plastid/chloroplast biogenesis (middle), shown on scales representing the physical position along the leaf, the calculated cell age and the sampling strategy employed by this study. Bar thickness approximately represents the measured magnitude of the process or the average expression level of the participating genes. The bottom panel represents the region of predicted action of the known candidate transcriptional regulators (with one negative regulator distinguished by a gray box), and highlights their absence at the initial stage

It is possible, considering both published data and our analyses, some of which place them in a global leaf developmental context at high resolution, and some of which provide completely novel insights, to recount a cellular life history which accounts for the events encompassing proliferation, differentiation and the development of chloroplasts (Fig. 7). In meristematic cells recruited into leaf primordia in the shoot apex, the cellular resources are predominantly invested into transcriptional regulation, cell proliferation and protein synthesis to drive cytoplasmic growth. The cell cycle is most active in these cells, and it operates at essentially its fastest possible rate. Once part of the elongating first leaf, major investment continues on transcriptional control processes and protein synthesis, as large-scale analyses observed in maize and rice [10, 11, 13] while proliferation of those progenitor cells, previously quantified in wheat and barley [5, 6, 14] gradually ceases within 24 h, in the first 10 mm at the leaf base. During this period, only one to two rounds of cell cycling take place on average. In these meristematic cells, very small proplastids are present and are proliferating extraordinarily rapidly and, in accordance with previous observations, both plastidial genome copies [14] and ribosomes [5, 11] accumulate to detectable levels.

The transition from cell cycling to cell expansion and plastid growth can be described as a transition from proliferation to cell differentiation (Fig. 7). It coincides remarkably well with the loss of phosphorylation of the core cell cycle negative regulator, RBR. As cells exit the cell cycle after about 1 day (still between 5 and 10 mm), cell expansion and the first phase of organelle growth—the “plastid” phase—become very significant. Plastids continue proliferating very rapidly. For example, between samples 2 and 3, whose difference in midpoint cell age is estimated as 8 h, two rounds of organelle division took place (plastid number multiplied by four). This reminisces of the fact that plastid division shares many characteristics with cyanobacterial division, of much shorter duration than eukaryotic cell division. At this stage, plastids are replicating their DNA [5, 6] and also growing rapidly in individual size, presumably by importing cytoplasmically translated proteins for pre-photosynthetic functions [11]. Indeed, plastid protein translocon genes peak in expression at this time, even though their products will also continue to play an essential role later on. The nuclear-encoded chloroplast development regulators RCB [39] and GNC [45] are expressed at this time; however, computational network analysis pointed also to an early role for a GLK homolog. Transcription in plastids, primarily of genetic machinery components as observed in barley and maize [7, 9], is first carried out by the nuclear-encoded RPOTp—any assembled plastid-encoded polymerase awaiting until the subsequent incorporation of its early sigma factors—at a time when their ribosomal RNAs are also rapidly building.

After 20 mm, between 1 and 1.5 days, when the exit from the cell cycle and the transition to differentiation is complete (Fig. 7), cells have already achieved nearly 50% of their final size, the peak in the plastid number signals the end of their division, but the “plastid build-up” phase continues unabated. Individual plastids are less than 20% of their final size, and greening only at 10% of its final value. As cells increase in size and remodel their cell walls, copies of the plastid genome and plastid ribosomes continue to accumulate rapidly, consistent with observations in maize showing chloroplast translation becomes a more substantial component of total cellular translation capacity [9]. Nevertheless, plastid growth decelerates. Nuclear regulatory function was computationally predicted for the light signalling-related HEMERA [43] and HY5 [42], while action of one PIF3 homolog expressed early might act here to prevent the premature initiation of greening [52, 53] unless under full light exposure. Expression data indicate that the primary role in plastid transcription of the nucleus-encoded RPOTp is gradually replaced by the plastid-encoded polymerase under the control of early sigma factors. Here forth, both plastid RNA polymerases act simultaneously, albeit the primary role is carried out by the plastid-encoded polymerase, as confirmed by observations in barley [38]. This is probably the point of highest overall plastid transcription capacity [6, 9], with transcripts for genetic machinery components further being preferentially translated [9].

As cells reach between 30 and 35 mm, less than 2 days after they became part of the developing leaf (Fig. 7), plastid DNA and ribosomes have reached more than 50% of their maximum value, but plastid growth becomes minimal. This sees the end of the plastid growth phase. The number of plastids per cell at this point shows a gradual, small but consistent decrease, which we cannot at present explain. This can be considered a transition stage, characterized by the intermediate sigma factors and preceding the bulk of greening, as seen by global analyses in maize and rice [10, 11, 13]. Interestingly, loss of the Arabidopsis homologs of only the intermediate wheat sigma factors, SIG2_2 and SIG6, peaking at the start of the chloroplast stage, results in reduced greening in Arabidopsis [59], while loss of other sigma factors can be compensated for. Also interestingly, this is the stage of peak expression of the plastid envelope-bound SP1 ubiquitin ligase, which remodels the outer envelope translocon complexes, of HY5, and of GUN1, involved in reporting plastid status to the nucleus.

Post-transition, over the following 2 days, the bulk of cellular activity appears focused towards developing the photosynthetic apparatus, a “chloroplast build-up” phase [10–13]. Three quarters of chloroplast growth and 85% of the total greening occur at this time. Chloroplast transcription is associated with expression of late sigma factors and produces [7, 9] and preferentially translates photosynthetic transcripts [9], as observed by analysis of barley and maize. It is noteworthy that such late sigma factors are those which respond to blue light (SIG5) or redox signals (SIG1) modulating photosynthetic chloroplast composition in response to the environment [60]. At the same time, thylakoid and photosynthetic component transcripts of nuclear-encoded genes also rapidly accumulate, their expression coinciding with that of GLK [41, 61] transcription factors.

To support or rule out the distinct stages of chloroplast biogenesis, we examined the levels of proteins that can be linked to the “plastid build-up” (proliferation, establishment of genetic machinery, establishment of protein import capacity) and the “chloroplast build-up” (photosynthetic development) phases. Of the six proteins selected, five exhibited profiles which closely matched those of their transcripts, and one continued to be present, and presumably active in protein import, as greening occurred, even though its transcript levels decreased. Overall, the protein profiles were consistent with the organelle developmental phases based on transcript accumulation profiles. This is not surprising, given that good correlations have been previously observed between transcript and “weighted” protein profiles for developmental or photosynthetic functions [62, 63].

### Regulatory genes of organelle development

Having established distinct phases and underlying processes contributing to chloroplast build-up, we set out to link these to regulators. Recently, a very large gene regulatory network was generated using the GENIE3 algorithm [54], based on transcription profiles of 800 samples from wheat [17]. We examined a subnetwork of this that included only plastid-targeted proteins, but failed to observe substantial links with our selected regulatory genes, for which there is prior evidence for plastid development functions. This highlights the limited suitability of existing gene expression profile data (largely focused on adult organs or on optimal or stress conditions) to link our cohorts of genes with their regulators. Identification of transcription factors capable of acting as “master switches” of chloroplast development [8, 64] would constitute an achievement of far-reaching consequences and is one of our ultimate experimental goals. Expression profiles such as those we generated here, which represent a timeline of combined cellular histories, might represent a better approach in the search for regulators. Our results, interestingly, suggested substantial potential roles for transcriptional regulators previously identified through their involvement in light signalling, RCB and HEMERA, in addition to the GLKs, plus a negative role for PIF3, in the emerging leaf region in which light exposure, even under continuous light, could have been limited. They also highlighted our sparse knowledge of, particularly, the control mechanisms of early plastid development. Other than the factors referred above, CIA2 [47] was identified through its role in the expression of protein import genes and further found to activate ribosomal components. Of two CIA2 homologs in wheat, one exhibited early expression coinciding with the onset of the plastid growth phase, although computational approaches predicted connectivity to very few genes for plastid-targeted proteins at this stage. The algorithm predicted connectivity of one GLK1 to more genes but of weak strength at this stage. A *cia2* Arabidopsis mutant shows only a mild phenotype, which suggests that master regulators initiating chloroplast development by triggering plastid build-up almost certainly await identification.

In a global context, a combination of stagnating yield increases, rising population, changing diets, expanding climate-related environmental stresses and the need to limit environmental impacts of agricultural inputs, all combine to create a perfect storm for future human food supply [65]. It has been argued that an improvement of photosynthetic yield, even by synthetic means, is one of the few remaining strategies for tackling this enormous challenge [66]. Understanding the development of the photosynthetic apparatus not only will unravel a fundamental biological process, it is also essential to address such a challenge. The data we present here could help accelerate achieving this goal.

## Methods

### Plant material and growth conditions

*Triticum aestivum* var. Chinese Spring WPGS 6265, line L42 (John Innes Centre Germplasm Resources Unit) was utilized. Seeds of similar size were surface sterilized for 10 min with 90% ethanol, rinsed 5 times with sterile distilled water. They were stratified for 3 days at 4^o^C, placed on soil and covered with a 1-cm top layer of vermiculite. Seedlings were grown at 23 °C, 60% relative humidity under constant light (285–395 μmol m^−2^ s^−1^) provided by a combination of white fluorescent lamps (TLD 840), red and blue LED (Sonlight, Italy).

### Sample collection

Samples were collected from 6-day-old seedlings at a time when the first leaf (170 mm) showed no sheath and a barely detectable transparent ligule at ca. 8 mm from the leaf attachment point at the base. The seedlings were detached right at the point of seed emergence, and the enclosing coleoptile (35 mm) was removed by gently unrolling, with care to not damage leaf 1. Similarly, leaf 1 was separated from the developing second leaf (85 mm) which had enclosed an emerging leaf 3 primordium (ca. 1.5 mm in length). The emerging leaf 3, embedded plastochrons 1 and 2 and the shoot apical meristem (ca. 300 μm in height) combined served as sample 1. The above steps were carried out under a stereomicroscope (SMZ-2 T, Nikon, Japan). Leaf primordia and shoot apical meristem were observed using Nikon SMZ-1500 and Leica (Switzerland) EZ4 HD microscopes. The 6-day-old leaf 1 produced 13 samples (sections 2 to 14) covering various developmental stages of which 2 to 9 contained consecutive 5 -mm sections, while samples 10 to 14 were discontinuous and 10 mm each. A fully mature sample 15 (20 mm) was collected from the midpoint of 14-day-old leaf 1, grown from the same batch, shortly before senescence began to appear at the leaf tip.

### Cell cycle analysis

Plant tissue was finely chopped using Wilkinson blades by adding an appropriate volume of lysis buffer (Partec cystain UV precise P Solution 1, Sysmex, Germany) and stained with DAPI (Partec cystain UV precise P Solution 2). The lysis mixture was passed through a 20–30-μm filter (CellTrics, Sysmex) and run through the flow cytometer (Sysmex CyFlow® Space, Sysmex). DNA content (2C or 4C) was determined by counting a minimum of 10,000 nuclei per sample. Frequency histogram outcome was fit into the cell cycle analysis tool of the instrument’s software to obtain the percentages of cell cycle phases.

### Cellular microscopy

Chloroplast quantitation was performed by modifying the previously published protocol [67]. The leaf samples were fixed (3.5% (v/v) Glutaraldehyde and 0.05% (v/v) Tween 20) for 1 h in the dark including vacuum infiltration for 5 min at 500 mBar (DNA Mini vacuum centrifuge) after every 30 min. The fixative was replaced with EDTA solution (100 mM, pH 9), and samples were incubated overnight at 65 °C and stored at 4 °C. Microscopy slides were prepared with a small amount of tissue selected from the midpoint of every section and mounted in 50% (v/v) glycerol. The mesophyll cells were observed under a Nikon Optiphot-2 microscope, focussed under the × 40 (Plan Fluor) objective to perform quantitative measurements using Nomarski optics. Data were collected live at × 40 magnification using a MicroPublisher 5.0 RTV (QImaging, Canada) camera and NIS-Elements AR 2.30 (Nikon) software. The cell size, plastid size and count were determined live by the inbuilt area and count tools. Total chloroplasts were deduced by live counting on different planes, completely moving out of focus and slowly focussing inwards (until the last plane, losing the focus again) marking only visible plastids in different planes [30]. We noted this method to yield a higher total number of plastids than those observed at a single plane by captured, single photographs [14, 16].

Fluorescence microscopy samples were fixed as above and immersed (2 min) in the DNA-binding 4′,6-diamidino-2-phenylindole (DAPI, Partec cystain UV precise P Solution 2) dye and mounted in the same solution. Mesophyll cells were visualized under a Nikon H600L Eclipse 80i fluorescence microscope, using × 60 Plan Apo oil immersion objective, UV excitation and blue emission filters.

### Extraction of nucleic acids

The DNA and RNA were simultaneously extracted from the same samples using NucleoSpin kit (Macherey Nagel, Germany). The frozen wheat sample was homogenized in two steps with a metal pestle fixed to a mechanical drill and Ultra Turrax (IKA, UK) homogenizer after adding the lysis buffer. The concentration of purified DNA was measured using the NanoDrop™ 1000 Spectrophotometer (Thermo, UK).

### Chloroplast genome quantitation

Chloroplast genome copy numbers were determined by quantitative PCR (Rotor-Gene Q real-time PCR cycler, Qiagen, UK) using the SyGreen Mix Lo-ROX (PCRBiosystems, UK). Primers (Additional file 5: Table S5) used for the quantitation of plastid genomes were designed in the matching sequence of two available wheat chloroplast genomes [68, 69]. Standard curves were prepared with known concentrations (25 pg/μl to 0.0025 pg/μl) of purified (QIAquick kit, Qiagen) PCR-amplified products representing two nuclear single-copy genes—*TaKO1* and *TaKS* (of A, B and D genomes selected from NCBI, Ensemble Plants and aligned in Clustal Omega) and three chloroplast genes (*rbcL*—large single-copy region, *ndhD*—small single-copy region and *rps7*—inverted repeat region, Additional file 1: Figure S6). The qPCR was performed with the DNA samples (diluted 10-fold for nuclear genes and 100-fold for the plastid genes) alongside the standards. Absolute plastid DNA copies were measured per haploid nuclear genome against standard curves of purified PCR products for each target gene by taking the mean values of three chloroplast genes and two nuclear single-copy genes [(*rbcL* + *ndhD* + (*rps7* / 2)) / 3] / [(*TaKO1* + *TaKS*) / 2].

### RNA quality

The concentration and quality of RNA were measured with an Agilent (UK) 2100 Bioanalyzer and Agilent Expert software [70]. A total of 7 μg high-quality RNA (RNA integrity number 8-10) was mixed with the matrix of RNA stable tubes (Sigma) and dried at room temperature by vacuum centrifuge for 2–3 h. Dry RNA tubes were left in a desiccator with silica gel for 2 days, sealed in moisture barrier bags (Sigma) and submitted for sequencing.

### RNA sequencing

The RNA-Seq analysis was performed with three biological replicates per sample: sample 1-sample 15 (see “Sample collection”). The libraries for stranded RNA sequencing were constructed using the TruSeq Stranded mRNA Sample Preparation Kit (Illumina) according to the manufacturer’s instructions and assessed using an Agilent (UK) 2100 Bioanalyzer. The library clonal clusters were generated by using a cBot with the TruSeq PE Cluster Kit (Illumina), and sequenced by using the TruSeq SBS Kit (Illumina) and the paired-end sequencing method to obtain 2 × 100 bp paired-end reads. The RNA-seq reads were quality checked and trimmed by using Trimmomatic (v0.36) [71] with the LEADING:20, TRAILING:20 and MINLEN:36 parameters, and mapped to the reference sequence assembly of Chinese Spring bread wheat (IWGSC RefSeq v1.0 assembly) [19] with bwa-mem (0.7.17-r1188) [72]. The read counts data were generated using featureCounts (v.1.5) (http://bioinf.wehi.edu.au/featureCounts/) with the HC dataset of IWGSC Refseq v1.1 annotation (iwgsc_refseqv1.1_genes_2017July06), and normalized based on reads per million (RPM).

### Transcriptome analysis

Wheat genes representing RPM ≥ 1 in all three biological replicates from at least one sampling time were defined as expressed genes; those genes were used to assess the transcriptome differences across the samples by a principal component analysis (PCA) plot. Top and bottom 5% of genes sorted by factor loading of each principal component in the PCA were used in gene set enrichment analysis (GSEA) with the gene ontology (GO) terms, and the GSEA results were summarized by using the REVIGO web server. Based on the averaged RPM of three biological replicates, wheat genes with RPM ≥ 5 for at least one sample, and a fold change during the time course (maximum RPM/minimum RPM) ≥ 2, and coefficient of variation ≥ 0.2 were defined as dynamically expressed genes (DYGs) in the transcriptome dataset; the *Z*-scored RPM data of the DYGs were used for co-expressed gene analysis by using weighted correlation network analysis (WGCNA (v.1.63)) with soft threshold = 14. The gene sets of each WGCNA-based co-expressed gene module were used for gene set functional enrichment analysis based on the functional annotation of the closest homologs in Arabidopsis [22] by the hypergeometric test using the phyper function of R.

### Functional gene annotation

The representative deduced protein sequences in the HC dataset of IWGSC Refseq v1.1 annotation were used to search for their closest homologs in Arabidopsis genes by identifying the highest hit of BLASTP search (e-value < 1e−5) against the representative protein dataset in TAIR10. The chloroplast protein genes in wheat were predicted based on the homology to Arabidopsis genes whose annotation of subcellular localization is “plastid” by any of the following: “predicted by SUBA consensus” or “experimentally inferred by fluorescent protein and simultaneously plastid protein location according to either TAIR10, SwisProt or AmiGO” or “experimentally inferred by mass spectrometry by at least two different experiments” in the SUBA4 database (https://suba.live/aboutSUBA4.html). Overrepresented functional class GO terms in each module was assessed as that of the corresponding Arabidopsis homologs using BINGO [73].

### Gene regulatory network inference

Gene regulatory network (GRN) inference was performed using the R package GENIE3 [54], which can infer GRNs using a decision tree-based machine learning algorithm based on gene expression data. The GRN associated with chloroplast-related genes in wheat was constructed with the expression datasets of the DYGs of wheat genes homologous to the chloroplast-localized Arabidopsis genes as targets and those homologous to CIA2, GLK1, GNC, HY5, RCB/SVR4, NCP and HEMERA in Arabidopsis, and to PIFs in rice and maize, as regulators.

### Reverse transcription and plastid ribosomal build-up

The total RNA (1 μg) was reverse-transcribed using Maxima cDNA synthesis kit (Thermo Scientific) by adding a specific primer (0.1 pM) that recognizes both 16S rRNA (plastid rRNA) and 18S rRNA (cytosolic rRNA), along with the kit’s oligo-dT and random hexamers to ensure unbiased reverse transcription. The cDNA samples were run alongside purified PCR products used as standards (over four orders of magnitude). The ratio of absolute amount of 16S rRNA and 18S rRNA was used to measure the plastid ribosomal build-up. The primers used for reverse transcription and PCR/ qPCR analysis are listed in Additional file 5: Table S5. In addition, the plastid ribosomal build-up was also analyzed by the area of RNA electropherogram peaks (measured on ImageJ) produced by the Bioanalyzer. The ratio of total 16S rRNA and 18S rRNA peaks was used to quantify plastid ribosomal capacity (displayed in Fig. 4i). Both methods of plastid rRNA quantitation provided very similar results, demonstrating that the majority of organellar, total 16S rRNA is plastidic.

### Chlorophyll quantitation

Chlorophyll content along the developing leaf was measured following published methods [74] using Thermo Scientific Heλios-β spectrophotometer. The amount of chlorophyll (μmol) per gram fresh weight was measured in triplicate samples, each containing material from at least 5 leaves.

### Protein extraction and immunoblotting

Total proteins were extracted following a urea-based method followed by cold acetone precipitation [75]. Leaf tissue was homogenized with the extraction buffer (1% SDS, 8 M urea) and the protein was allowed to precipitate for 2 h in cold acetone (dried using molecular sieves 4A—Sigma—for at least the previous 24 h, at − 20 °C). The protein pellet was dissolved in 8 M urea solution (without SDS). Proteins were separated on the SDS-PAGE gel and blotted onto nitrocellulose or PVDF membranes. Immunoblotting was performed with the following primary and Alkaline phosphatase-conjugated secondary antibodies (Agrisera, Sweden): RCB/SVR4 (AS13 2725), DRP5B/ARC5 (AS12 2634), TIC40 (AS10 709), PsbO (AS06 142-33), LHCB1 (AS01 004), Histone 3 (AS15 2855) and goat-antirabbit secondary (AS09 607), or rabbit antichicken secondary (A9171, Sigma, UK). The primary antibody against SBPase has been described [76]. Antibodies were used at 1:2000 dilution except for AS01 004 and AS15 2855 (1:2500), in 5% blotto. AS12 2634 was prepared in 2% blotto and required long signal detection. The signals were detected using sigmafast BCIP®/NBT substrate solution (Sigma).

For RBR and P-RBR, protein extraction and immunoblotting and the antibodies used were as described [29], with one lane using Thermo Scientific™ PageRuler™ Plus Prestained Protein Ladder.

### Analysis of cellular and chloroplast quantitative data

The cellular quantitative data (cell area, chloroplast count and mean chloroplast area) were obtained from at least three biological replicate sample sets, the exact numbers indicated for each experiment. The details of the statistical calculations are listed in Additional file 5: Table S6, and the R package scripts for boxplots are listed in Additional file 5: Table S7. The effect of cell division on plastid counts per cell (one division halving the average number of plastids per cell) was accounted for by applying a correction factor. The correction factor was 2 (every cell doubles once) for the transition from sample 1 to 2, as described in the text. For the transition between subsequent samples, the factor was calculated from the measured proportion of nuclei in S phase, relative to that in sample 1, considering that, given the absence of endoreduplication, every cell undergoing S phase would subsequently undergo mitosis. Cell age for the midpoint of each sample was derived from published observations [15], corrected for the measured rate of leaf elongation in our samples (44, 43 and 42 mm/day on days 5, 6 and 7). Sample number, position, midpoint, calculated age and correction factors for cell division (when needed for parameters changing on a per cell basis) are shown on Additional file 5: Table S8.

## Supplementary Information

**Additional file 1. Figure S1 to Figure S9.**

**Additional file 2: Table S1.** Expression of all genes identified as dynamically expressed (DYGs). Absolute expression, average of three samples, measured as reads per kilobase per million bases (RPMs) and relative expression as Z-score. The closest homologs of maize, rice, *Brachypodium* and Arabidopsis are indicated, as is the expression module as identified by WGCNA.

**Additional file 3: Table S2.** Expression of all clustered genes representing individual functional classes, displayed in Additional file 1: Figure S2. Absolute (measured as RPMs) as well as relative (Z-score) expression are provided. For transcription, translation and protein fate classes, the subcellular localisation of the encoded protein is also provided.

**Additional file 4: Table S3.** Expression of all clustered hormone signature genes displayed in Additional file 1: Figure S3. Absolute (measured as RPMs) as well as relative (Z-score) expression are provided.

**Additional file 5: Table S4.** List of genes representative of key biological functions whose expression was individually plotted in Figs. 3, 4, 5, 6 and Additional file 1 Figures 8-9. **Table S5.** Primers used for genome copy number and rRNA quantitation. **Table S6.** Summary of quantitative cellular and other parameters and data analysis. **Table S7.** R script used to generate box plots. **Table S8.** Correction parameters for cell age and mitosis.

**Additional file 6: Table S9.** Expression of genes for each of the 12 modules of DYGs for chloroplast-targeted proteins, displayed in Fig. 6b. Absolute (RPMs) and relative (Z-score) expression are provided, as are overrepresented biological function gene ontology terms for each module.

**Additional file 7: Table S10.** Regulatory weighting estimated by GENIE3, displayed in Fig. 6c and Figure S9. The values represent the weighting of regulation between candidate transcriptional regulators and genes for chloroplast-targeted proteins.

**Additional file 8.** Review history.

## Data Availability

RNA-Seq data (45 individual biological samples) are deposited in DDBJ under BioProject accession PRJDB8633 [77].

## References

[1] Ort DR, Merchant SS, Alric J, Barkan A, Blankenship RE, Bock R, Croce R, Hanson MR, Hibberd JM, Long SP (2015). Redesigning photosynthesis to sustainably meet global food and bioenergy demand. Proc Natl Acad Sci.

[2] Avramova V, Sprangers K. Beemster GT. The maize leaf: another perspective on growth regulation. Trends Plant Sci. 2015;20(12):787–97. 10.1016/j.tplants.2015.09.002. 10.1016/j.tplants.2015.09.002 26490722

[3] Kuroiwa T, Suzuki T, Ogawa K, Kawano S. The chloroplast nucleus - distribution, number, size, and shape, and a model for the multiplication of the chloroplast genome during chloroplast development. Plant Cell Physiol. 1981;22:381–96.

[4] Leech RM, Rumsby MG, Thomson WW (1973). Plastid differentiation, acyl lipid, and fatty-acid changes in developing green maize leaves. Plant Physiol.

[5] Dean C, Leech RM (1982). Genome expression during normal leaf development: I. Cellular and chloroplast numbers and DNA, RNA, and protein levels in tissues of different ages within a seven-day-old wheat leaf. Plant Physiol.

[6] Baumgartner BJ, Rapp JC, Mullet JE (1989). Plastid transcription activity and DNA copy number increase early in barley chloroplast development. Plant Physiol.

[7] Baumgartner BJ, Rapp JC, Mullet JE. Plastid genes encoding the transcription translation apparatus are differentially transcribed early in barley (*Hordeum vulgare*) chloroplast development - evidence for selective stabilization of *psba* messenger-RNA. Plant Physiol. 1993;101(3):781–91. 10.1104/pp.101.3.781. 10.1104/pp.101.3.781 PMC158691 12231729

[8] Jarvis P, Lopez-Juez E (2013). Biogenesis and homeostasis of chloroplasts and other plastids. Nat Rev Mol Cell Biol.

[9] Chotewutmontri P, Barkan A (2016). Dynamics of chloroplast translation during chloroplast differentiation in maize. PLoS Genet.

[10] Li P, Ponnala L, Gandotra N, Wang L, Si Y, Tausta SL, Kebrom TH, Provart N, Patel R, Myers CR, Reidel EJ, Turgeon R, Liu P, Sun Q, Nelson T, Brutnell TP (2010). The developmental dynamics of the maize leaf transcriptome. Nat Genet.

[11] Majeran W, Friso G, Ponnala L, Connolly B, Huang M, Reidel E, et al. Structural and metabolic transitions of C4 leaf development and differentiation defined by microscopy and quantitative proteomics in maize. Plant Cell. 2010;22(11):3509–42. 10.1105/tpc.110.079764. 10.1105/tpc.110.079764 PMC3015116 21081695

[12] Pick TR, Bräutigam A, Schlüter U, Denton AK, Colmsee C, Scholz U, et al. Systems analysis of a maize leaf developmental gradient redefines the current C4 model and provides candidates for regulation. Plant Cell. 2011;23(12):4208–20. 10.1105/tpc.111.090324. 10.1105/tpc.111.090324 PMC3269860 22186372

[13] Wang L, Czedik-Eysenberg A, Mertz RA, Si Y, Tohge T, Nunes-Nesi A, Arrivault S, Dedow LK, Bryant DW, Zhou W, Xu J, Weissmann S, Studer A, Li P, Zhang C, LaRue T, Shao Y, Ding Z, Sun Q, Patel RV, Turgeon R, Zhu X, Provart NJ, Mockler TC, Fernie AR, Stitt M, Liu P, Brutnell TP (2014). Comparative analyses of C(4) and C(3) photosynthesis in developing leaves of maize and rice. Nat Biotechnol.

[14] Boffey SA, Ellis JR, Sellden G, Leech RM (1979). Chloroplast division and DNA synthesis in light-grown wheat leaves. Plant Physiol.

[15] Boffey SA, Selldén G, Leech RMJPP (1980). Influence of cell age on chlorophyll formation in light-grown and etiolated wheat seedlings. Plant Physiol.

[16] Pyke KA, Leech RM (1987). The control of chloroplast number in wheat mesophyll-cells. Planta.

[17] Ramirez-Gonzalez RH, Borrill P, Lang D, Harrington SA, Brinton J, Venturini L, Davey M, Jacobs J, van Ex F, Pasha A (2018). The transcriptional landscape of polyploid wheat. Science.

[18] Esau K (1977). Anatomy of seed plants.

[19] Appels R, Eversole K, Feuillet C, Keller B, Rogers J, Stein N, et al. Shifting the limits in wheat research and breeding using a fully annotated reference genome. Science. 2018;361:661–73. 10.1126/science.aar7191. 10.1126/science.aar7191 30115783

[20] Levin M, Anavy L, Cole AG, Winter E, Mostov N, Khair S, Senderovich N, Kovalev E, Silver DH, Feder M, Fernandez-Valverde SL, Nakanishi N, Simmons D, Simakov O, Larsson T, Liu SY, Jerafi-Vider A, Yaniv K, Ryan JF, Martindale MQ, Rink JC, Arendt D, Degnan SM, Degnan BM, Hashimshony T, Yanai I (2016). The mid-developmental transition and the evolution of animal body plans. Nature.

[21] Langfelder P, Horvath S (2008). WGCNA: an R package for weighted correlation network analysis. BMC Bioinformatics.

[22] López-Juez E, Dillon E, Magyar Z, Khan S, Hazeldine S, de Jager SM, Murray JA, Beemster GT, Bögre L, Shanahan H (2008). Distinct light-initiated gene expression and cell cycle programs in the shoot apex and cotyledons of Arabidopsis. Plant Cell.

[23] Avramova V, Sprangers K, Beemster GT (2015). The maize leaf: another perspective on growth regulation. Trends Plant Sci.

[24] López-Juez E, Bowyer JR, Sakai TJP (2007). Distinct leaf developmental and gene expression responses to light quantity depend on blue-photoreceptor or plastid-derived signals, and can occur in the absence of phototropins. Planta.

[25] Liu WY, Chang YM, Chen SC, Lu CH, Wu YH, Lu MY, Chen DR, Shih AC, Sheue CR, Huang HC (2013). Anatomical and transcriptional dynamics of maize embryonic leaves during seed germination. Proc Natl Acad Sci.

[26] Ivanov VB, Dubrovsky JG (1997). Estimation of the cell-cycle duration in the root apical meristem: a model of linkage between cell-cycle duration, rate of cell production, and rate of root growth. Int J Plant Sci.

[27] Mickelson-Young L, Wear E, Mulvaney P, Lee TJ, Szymanski ES, Allen G, Hanley-Bowdoin L, Thompson W (2016). A flow cytometric method for estimating S-phase duration in plants. J Exp Bot.

[28] De Veylder L, Beeckman T, Inze D (2007). The ins and outs of the plant cell cycle. Nat Rev Mol Cell Biol.

[29] Oszi E, Papdi C, Mohammed B, Petko-Szandtner A, Leviczky T, Molnar E, Galvan-Ampudia C, Khan S, Juez EL, Horvath B (2020). E2FB interacts with retinoblastoma related and regulates cell proliferation during leaf development. Plant Physiol.

[30] Pyke K. Analysis of plastid number, size, and distribution in Arabidopsis plants by light and fluorescence microscopy. In: Chloroplast Research in Arabidopsis: Springer; 2011. p. 19–32. 10.1007/978-1-61779-234-2_2. 10.1007/978-1-61779-234-2_2 21822830

[31] Liang Z, Zhu N, Mai KK, Liu Z, Tzeng D, Osteryoung KW, Zhong S, Staehelin LA, Kang BH (2018). Thylakoid-bound polysomes and a dynamin-related protein, FZL, mediate critical stages of the linear chloroplast biogenesis program in greening Arabidopsis cotyledons. Plant Cell.

[32] Gao H, Sage TL, Osteryoung KW (2006). FZL, an FZO-like protein in plants, is a determinant of thylakoid and chloroplast morphology. Proc Natl Acad Sci.

[33] Larkin RM, Stefano G, Ruckle ME, Stavoe AK, Sinkler CA, Brandizzi F, et al. Reduced chloroplast coverage genes from *Arabidopsis thaliana* help to establish the size of the chloroplast compartment. Proc Natl Acad Sci. 2016;113(8):E1116–25. 10.1073/pnas.1515741113. 10.1073/pnas.1515741113 PMC4776492 26862170

[34] Pfalz J, Pfannschmidt T (2013). Essential nucleoid proteins in early chloroplast development. Trends Plant Sci.

[35] Udy DB, Belcher S, Williams-Carrier R, Gualberto JM, Barkan A (2012). Effects of reduced chloroplast gene copy number on chloroplast gene expression in maize. Plant Physiol.

[36] Oldenburg DJ, Rowan BA, Zhao L, Walcher CL, Schleh M, Bendich AJ (2006). Loss or retention of chloroplast DNA in maize seedlings is affected by both light and genotype. Planta.

[37] Ling Q, Huang W, Baldwin A, Jarvis P (2012). Chloroplast biogenesis is regulated by direct action of the ubiquitin-proteasome system. Science.

[38] Zhelyazkova P, Sharma CM, Forstner KU, Liere K, Vogel J, Borner T (2012). The primary transcriptome of barley chloroplasts: numerous noncoding RNAs and the dominating role of the plastid-encoded RNA polymerase. Plant Cell.

[39] Yoo CY, Pasoreck EK, Wang H, Cao J, Blaha GM, Weigel D, Chen M (2019). Phytochrome activates the plastid-encoded RNA polymerase for chloroplast biogenesis via nucleus-to-plastid signaling. Nat Commun.

[40] Pribil M, Sandoval-Ibanez O, Xu WT, Sharma A, Labs M, Liu QP, Galgenmuller C, Schneider T, Wessels M, Matsubara S (2018). Fine-Tuning of Photosynthesis Requires CURVATURE THYLAKOID1-Mediated Thylakoid Plasticity. Plant Physiol.

[41] Waters MT, Wang P, Korkaric M, Capper RG, Saunders NJ. Langdale JA. GLK transcription factors coordinate expression of the photosynthetic apparatus in Arabidopsis. Plant Cell. 2009;21(4):1109–28. 10.1105/tpc.108.065250. 10.1105/tpc.108.065250 PMC2685620 19376934

[42] Oyama T, Shimura Y, Okada K. The Arabidopsis *HY5* gene encodes a bZIP protein that regulates stimulus-induced development of root and hypocotyl. Genes Dev. 1997;11(22):2983–95. 10.1101/gad.11.22.2983. 10.1101/gad.11.22.2983 PMC316701 9367981

[43] Chen M, Galvao RM, Li M, Burger B, Bugea J, Bolado J, Chory J (2010). Arabidopsis HEMERA/pTAC12 initiates photomorphogenesis by phytochromes. Cell.

[44] Yang EJ, Yoo CY, Liu J, Wang H, Cao J, Li FW, Pryer KM, Sun TP, Weigel D, Zhou P, Chen M (2019). NCP activates chloroplast transcription by controlling phytochrome-dependent dual nuclear and plastidial switches. Nat Commun.

[45] Zubo YO, Blakley IC, Franco-Zorrilla JM, Yamburenko MV, Solano R, Kieber JJ, Loraine AE, Schaller GE (2018). Coordination of chloroplast development through the action of the GNC and GLK transcription factor families. Plant Physiol.

[46] Kobayashi K, Baba S, Obayashi T, Sato M, Toyooka K, Keranen M, Aro EM, Fukaki H, Ohta H, Sugimoto K, Masuda T (2012). Regulation of root greening by light and auxin/cytokinin signaling in Arabidopsis. Plant Cell.

[47] Sun C-W, Huang Y-C, Chang H-YJPp (2009). CIA2 coordinately up-regulates protein import and synthesis in leaf chloroplasts. Plant Physiol.

[48] Wu GZ, Meyer EH, Richter AS, Schuster M, Ling Q, Schottler MA, Walther D, Zoschke R, Grimm B, Jarvis RP, Bock R (2019). Control of retrograde signalling by protein import and cytosolic folding stress. Nat Plants.

[49] Shikanai T, Fujii S (2013). Function of PPR proteins in plastid gene expression. RNA Biol.

[50] Rojas M, Ruwe H, Miranda RG, Zoschke R, Hase N, Schmitz-Linneweber C, Barkan A (2018). Unexpected functional versatility of the pentatricopeptide repeat proteins PGR3, PPR5 and PPR10. Nucleic Acids Res.

[51] Huq E, Al-Sady B, Hudson M, Kim CH, Apel M, Quail PH (2004). Phytochrome-interacting factor 1 is a critical bHLH regulator of chlorophyll biosynthesis. Science.

[52] Stephenson PG, Fankhauser C, Terry MJ (2009). PIF3 is a repressor of chloroplast development. Proc Natl Acad Sci.

[53] Lee N, Choi G (2017). Phytochrome-interacting factor from Arabidopsis to liverwort. Curr Opin Plant Biol.

[54] Huynh-Thu VA, Irrthum A, Wehenkel L, Geurts P (2010). Inferring regulatory networks from expression data using tree-based methods. PLoS One.

[55] Wang P, Kelly S, Fouracre JP, Langdale JA (2013). Genome-wide transcript analysis of early maize leaf development reveals gene cohorts associated with the differentiation of C4 Kranz anatomy. Plant J.

[56] Dubreuil C, Jin X, Barajas-Lopez JD, Hewitt TC, Tanz SK, Dobrenel T, Schroder WP, Hanson J, Pesquet E, Gronlund A (2018). Establishment of photosynthesis through chloroplast development is controlled by two distinct regulatory phases. Plant Physiol.

[57] Pipitone R, Eicke S, Pfister B, Glauser G, Falconet D, Uwizeye C, et al. A multifaceted analysis reveals two distinct phases of chloroplast biogenesis during de-etiolation in Arabidopsis. Elife. 2021;10. 10.7554/eLife.62709. 10.7554/eLife.62709 PMC7906606 33629953

[58] Kusumi K, Chono Y, Shimada H, Gotoh E, Tsuyama M, Iba K (2010). Chloroplast biogenesis during the early stage of leaf development in rice. Plant Biotechnol.

[59] Woodson JD, Perez-Ruiz JM, Schmitz RJ, Ecker JR, Chory J (2013). Sigma factor-mediated plastid retrograde signals control nuclear gene expression. Plant J.

[60] Puthiyaveetil S, McKenzie SD, Kayanja GE, Ibrahim IM (1864). Transcription initiation as a control point in plastid gene expression. Biochim Biophys Acta Gene Regul Mech.

[61] Fitter DW, Martin DJ, Copley MJ, Scotland RW, Langdale JA (2002). GLK gene pairs regulate chloroplast development in diverse plant species. Plant J.

[62] Ponnala L, Wang Y, Sun Q, van Wijk KJ (2014). Correlation of mRNA and protein abundance in the developing maize leaf. Plant J.

[63] Walley JW, Sartor RC, Shen Z, Schmitz RJ, Wu KJ, Urich MA, Nery JR, Smith LG, Schnable JC, Ecker JR, Briggs SP (2016). Integration of omic networks in a developmental atlas of maize. Science.

[64] Wang P, Hendron RW, Kelly S (2017). Transcriptional control of photosynthetic capacity: conservation and divergence from Arabidopsis to rice. New Phytol.

[65] Godfray HCJ, Beddington JR, Crute IR, Haddad L, Lawrence D, Muir JF, Pretty J, Robinson S, Thomas SM, Toulmin C (2010). Food security: the challenge of feeding 9 billion people. Science.

[66] Long SP, Marshall-Colon A, Zhu X-G (2015). Meeting the global food demand of the future by engineering crop photosynthesis and yield potential. Cell.

[67] Loudya N, Okunola T, He J, Jarvis P, Lopez-Juez E (2020). Retrograde signalling in a virescent mutant triggers an anterograde delay of chloroplast biogenesis that requires GUN1 and is essential for survival. Philos Trans R Soc Lond B Biol Sci.

[68] Middleton CP, Senerchia N, Stein N, Akhunov ED, Keller B, Wicker T, Kilian B (2014). Sequencing of chloroplast genomes from wheat, barley, rye and their relatives provides a detailed insight into the evolution of the Triticeae tribe. PLoS One.

[69] Ogihara Y, Isono K, Kojima T, Endo A, Hanaoka M, Shiina T, Terachi T, Utsugi S, Murata M, Mori N, Takumi S, Ikeo K, Gojobori T, Murai R, Murai K, Matsuoka Y, Ohnishi Y, Tajiri H, Tsunewaki K (2002). Structural features of a wheat plastome as revealed by complete sequencing of chloroplast DNA. Mol Genet Genomics.

[70] Schroeder A, Mueller O, Stocker S, Salowsky R, Leiber M, Gassmann M, Lightfoot S, Menzel W, Granzow M, Ragg T (2006). The RIN: an RNA integrity number for assigning integrity values to RNA measurements. BMC Mol Biol.

[71] Bolger AM, Lohse M, Usadel B (2014). Trimmomatic: a flexible trimmer for Illumina sequence data. Bioinformatics.

[72] Li H, Durbin R (2010). Fast and accurate long-read alignment with Burrows-Wheeler transform. Bioinformatics.

[73] Maere S, Heymans K, Kuiper M (2005). BiNGO: a Cytoscape plugin to assess overrepresentation of gene ontology categories in biological networks. Bioinformatics.

[74] Porra R, Thompson W, Kriedemann P. Determination of accurate extinction coefficients and simultaneous equations for assaying chlorophylls a and b extracted with four different solvents: verification of the concentration of chlorophyll standards by atomic absorption spectroscopy. Biochim Biophys Acta Bioenerg. 1989;975:384–94. 10.1016/S0005-2728(89)80347-0.

[75] López-Juez E, Hughes MJG (1995). Effect of blue light and red light on the control of chloroplast acclimation of light-grown pea leaves to increased fluence rates. Photochem Photobiol.

[76] Lefebvre S, Lawson T, Zakhleniuk OV, Lloyd JC, Raines CA, Fryer M (2005). Increased sedoheptulose-1,7-bisphosphatase activity in transgenic tobacco plants stimulates photosynthesis and growth from an early stage in development. Plant Physiol.

[77] Loudya N, Mishra P, Takahagi K, Uehara-Yamaguchi Y, Inoue K, Bogre L, Mochida K, López-Juez E: A gene expression map of the developing wheat leaf. Raw sequence reads. DNA DataBase of Japan. 2021. https://www.ncbi.nlm.nih.gov/bioproject/PRJDB8633/. 10.1186/s13059-021-02366-3 PMC8111775 33975629

