# Supplementary material for: Cellular and transcriptomic analyses reveal two-staged chloroplast biogenesis underpinning photosynthesis build-up in the wheat leaf
Source: Genome Biol. 2021 May 11;22:151. doi: 10.1186/s13059-021-02366-3 (PMC8111775; doi:10.1186/s13059-021-02366-3)
Supplement: Supplementary file 1 — Additional file 1. Figure S1 to Figure S9. [file 13059_2021_2366_MOESM1_ESM.pptx]

## Slide 1
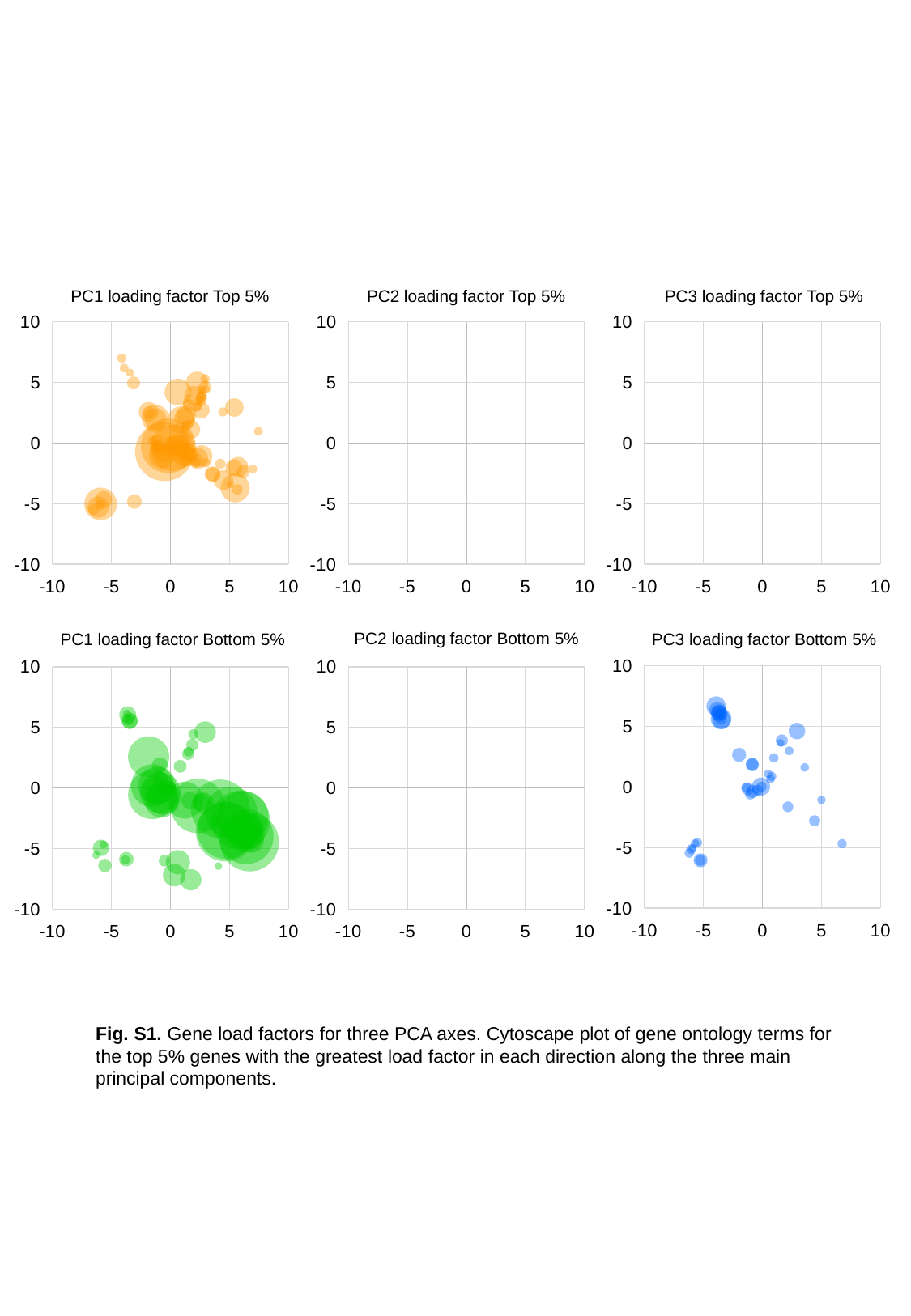

PC1 loading factor Top 5%
PC2 loading factor Top 5%
PC3 loading factor Top 5%
### Chart
| Category | |
|---|---|
### Chart
| Category | |
|---|---|
### Chart
| Category | |
|---|---|PC2 loading factor Bottom 5%
PC1 loading factor Bottom 5%
PC3 loading factor Bottom 5%
### Chart
| Category | |
|---|---|
### Chart
| Category | |
|---|---|
### Chart
| Category | |
|---|---|Fig. S1. Gene load factors for three PCA axes. Cytoscape plot of gene ontology terms for the top 5% genes with the greatest load factor in each direction along the three main principal components.

## Slide 2
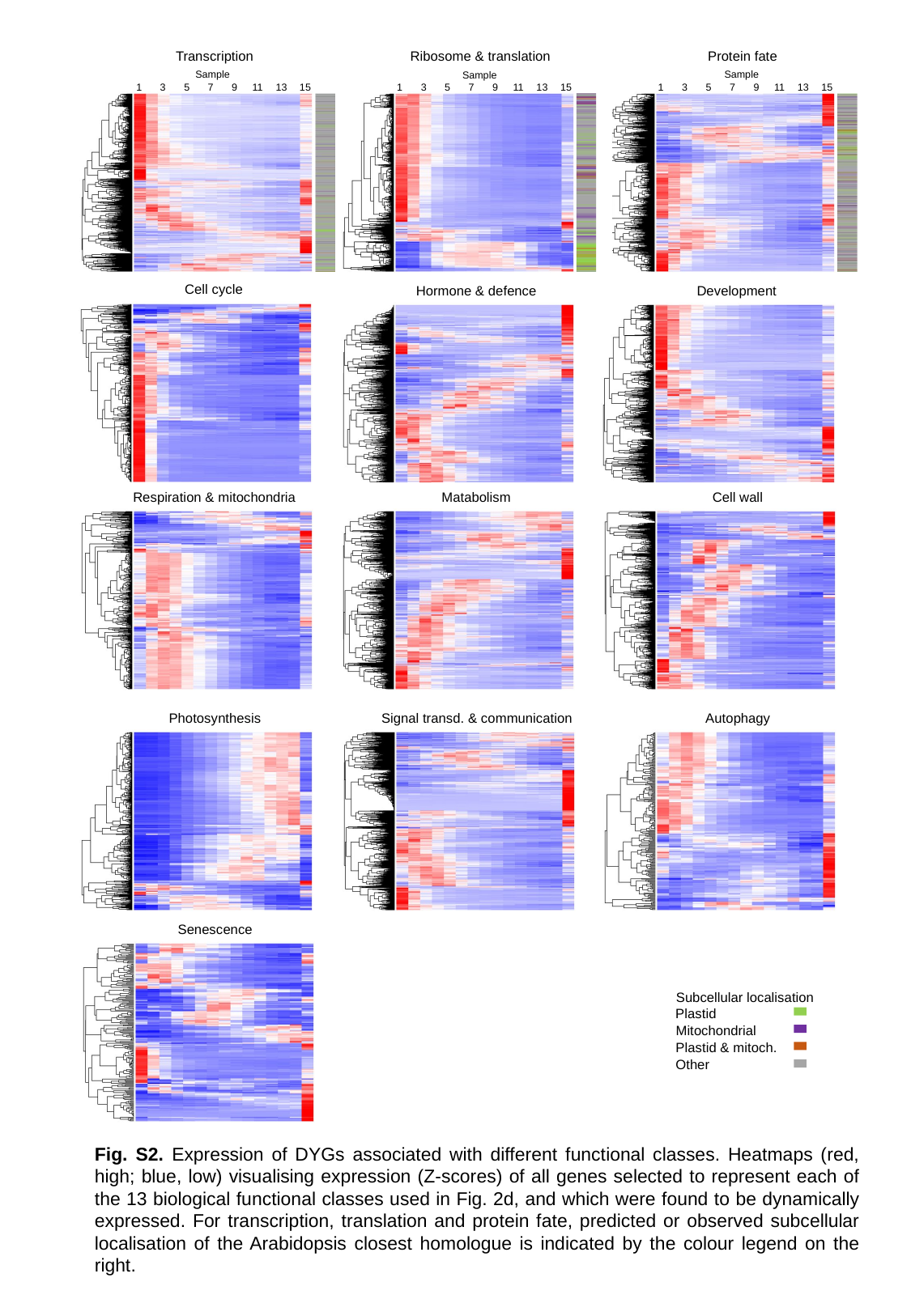

Transcription
Ribosome & translation
Protein fate
Sample
Sample
Sample
1
3
5
7
9
11
13
15
1
3
5
7
9
11
13
15
1
3
5
7
9
11
13
15
Cell cycle
Hormone & defence
Development
Matabolism
Cell wall
Respiration & mitochondria
Photosynthesis
Signal transd. & communication
Autophagy
Senescence
Subcellular localisation
Plastid
Mitochondrial
Plastid & mitoch.
Other
Fig. S2. Expression of DYGs associated with different functional classes. Heatmaps (red, high; blue, low) visualising expression (Z-scores) of all genes selected to represent each of the 13 biological functional classes used in Fig. 2d, and which were found to be dynamically expressed. For transcription, translation and protein fate, predicted or observed subcellular localisation of the Arabidopsis closest homologue is indicated by the colour legend on the right.

## Slide 3
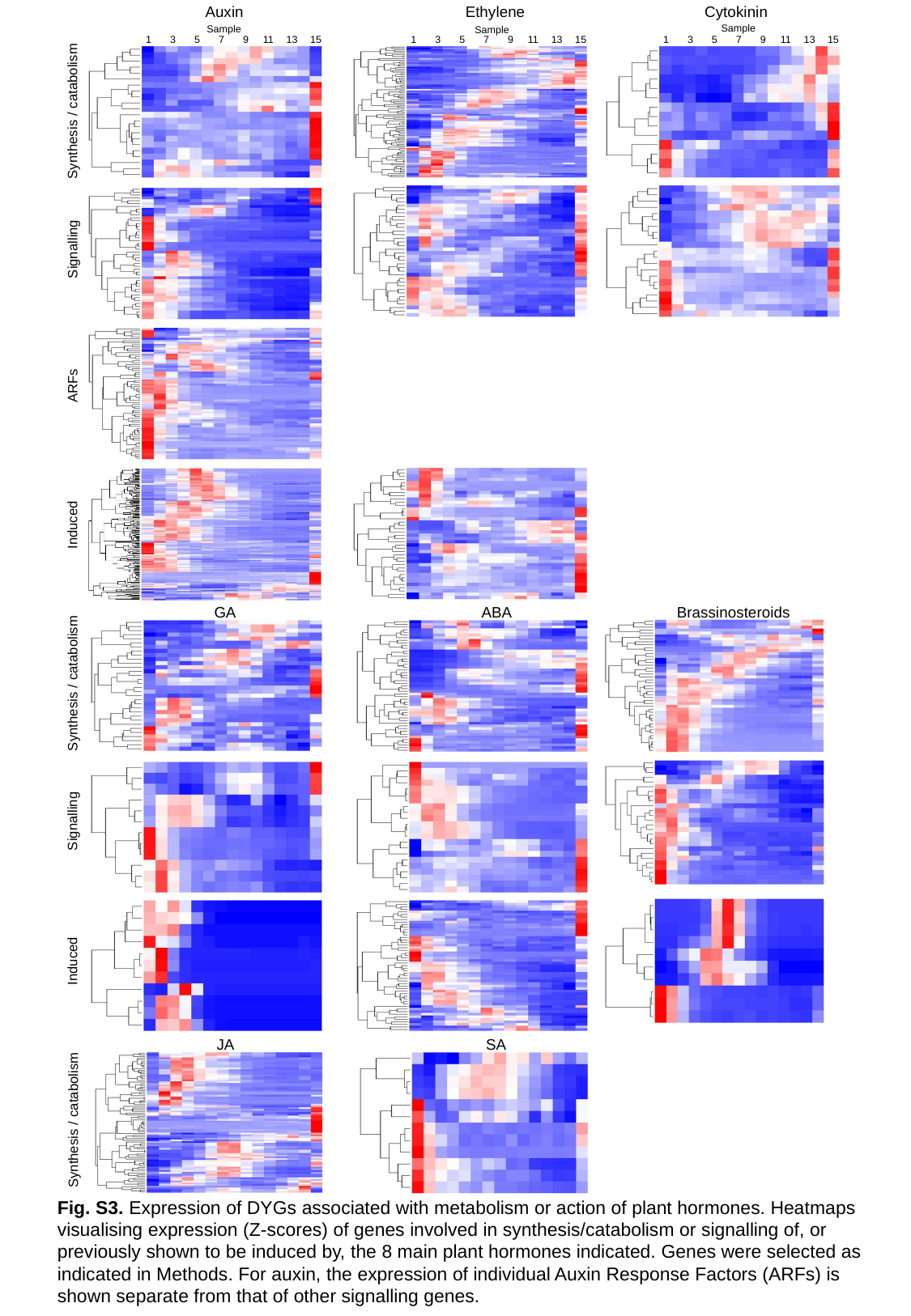

Auxin
Ethylene
Cytokinin
Sample
Sample
Sample
1
3
5
7
9
11
13
15
1
3
5
7
9
11
13
15
1
3
5
7
9
11
13
15
Synthesis / catabolism
Signalling
ARFs
Induced
GA
ABA
Brassinosteroids
Synthesis / catabolism
Signalling
Induced
JA
SA
Synthesis / catabolism
Fig. S3. Expression of DYGs associated with metabolism or action of plant hormones. Heatmaps visualising expression (Z-scores) of genes involved in synthesis/catabolism or signalling of, or previously shown to be induced by, the 8 main plant hormones indicated. Genes were selected as indicated in Methods. For auxin, the expression of individual Auxin Response Factors (ARFs) is shown separate from that of other signalling genes.

## Slide 4
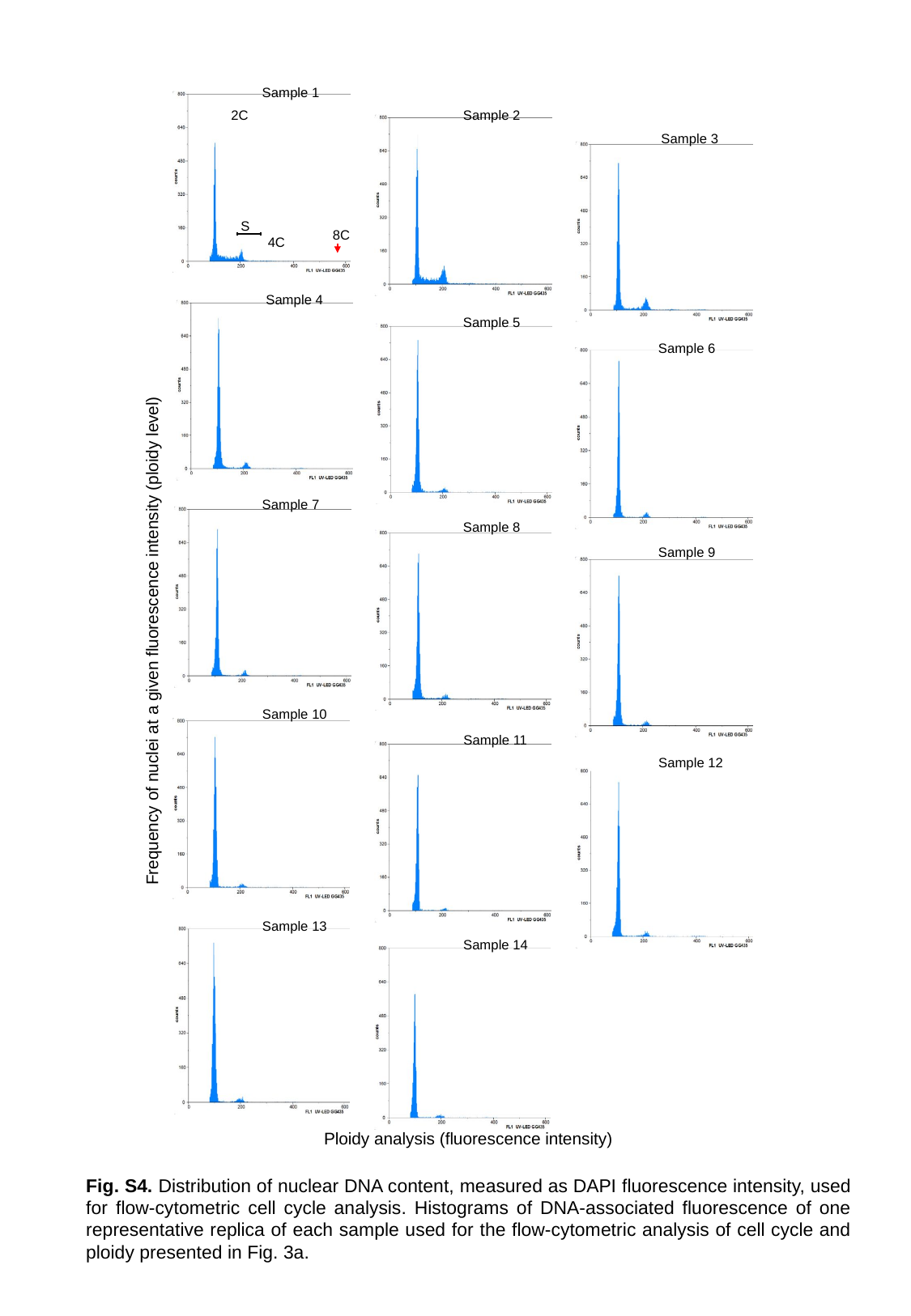

Sample 1
2C
Sample 2
Sample 3
S
8C
4C
Sample 4
Sample 5
Sample 6
Sample 7
Sample 8
Sample 9
Frequency of nuclei at a given fluorescence intensity (ploidy level)
Sample 10
Sample 11
Sample 12
Sample 13
Sample 14
Ploidy analysis (fluorescence intensity)
Fig. S4. Distribution of nuclear DNA content, measured as DAPI fluorescence intensity, used for flow-cytometric cell cycle analysis. Histograms of DNA-associated fluorescence of one representative replica of each sample used for the flow-cytometric analysis of cell cycle and ploidy presented in Fig. 3a.

## Slide 5
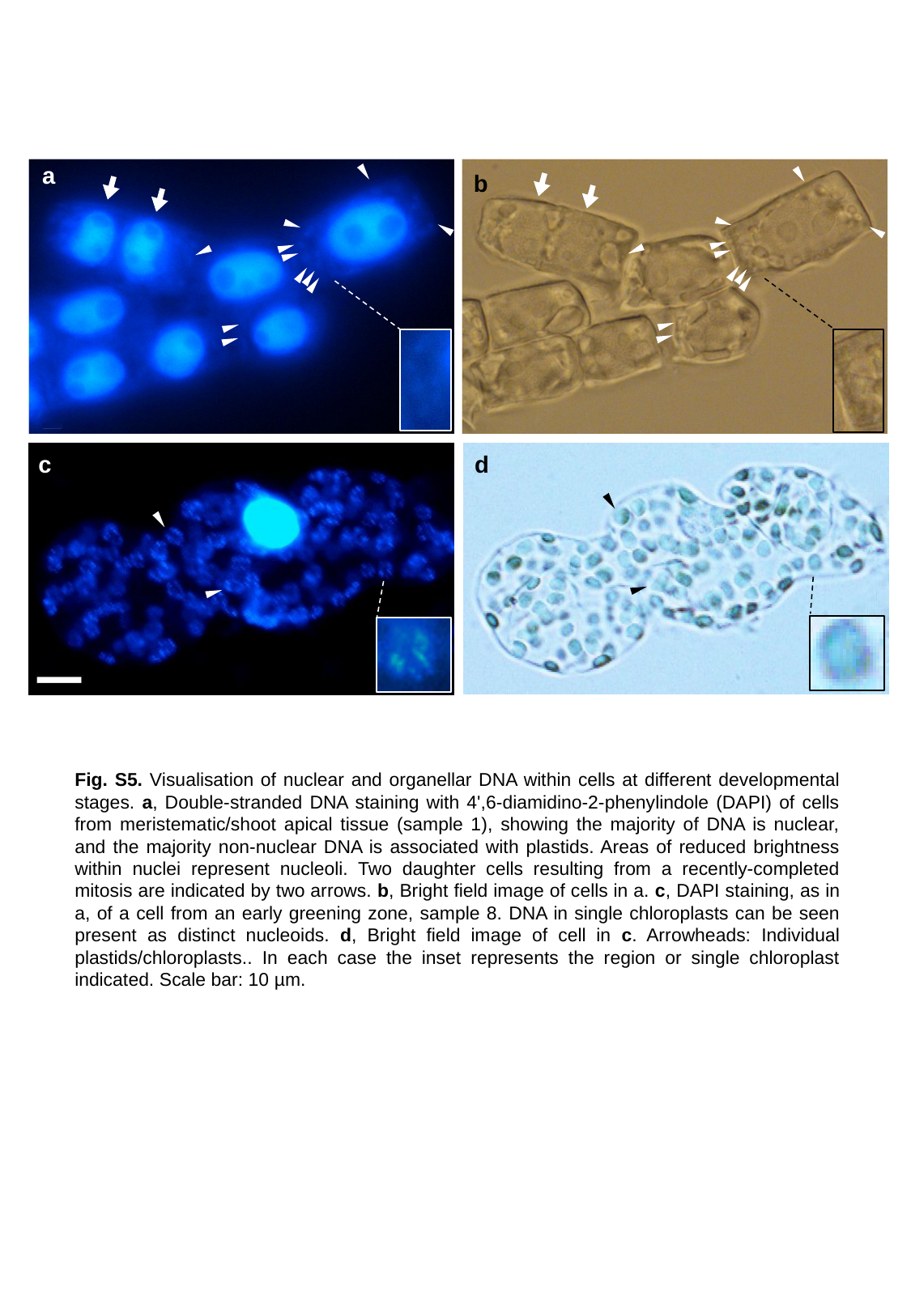

a
b
d
c
Fig. S5. Visualisation of nuclear and organellar DNA within cells at different developmental stages. a, Double-stranded DNA staining with 4',6-diamidino-2-phenylindole (DAPI) of cells from meristematic/shoot apical tissue (sample 1), showing the majority of DNA is nuclear, and the majority non-nuclear DNA is associated with plastids. Areas of reduced brightness within nuclei represent nucleoli. Two daughter cells resulting from a recently-completed mitosis are indicated by two arrows. b, Bright field image of cells in a. c, DAPI staining, as in a, of a cell from an early greening zone, sample 8. DNA in single chloroplasts can be seen present as distinct nucleoids. d, Bright field image of cell in c. Arrowheads: Individual plastids/chloroplasts.. In each case the inset represents the region or single chloroplast indicated. Scale bar: 10 µm.

## Slide 6
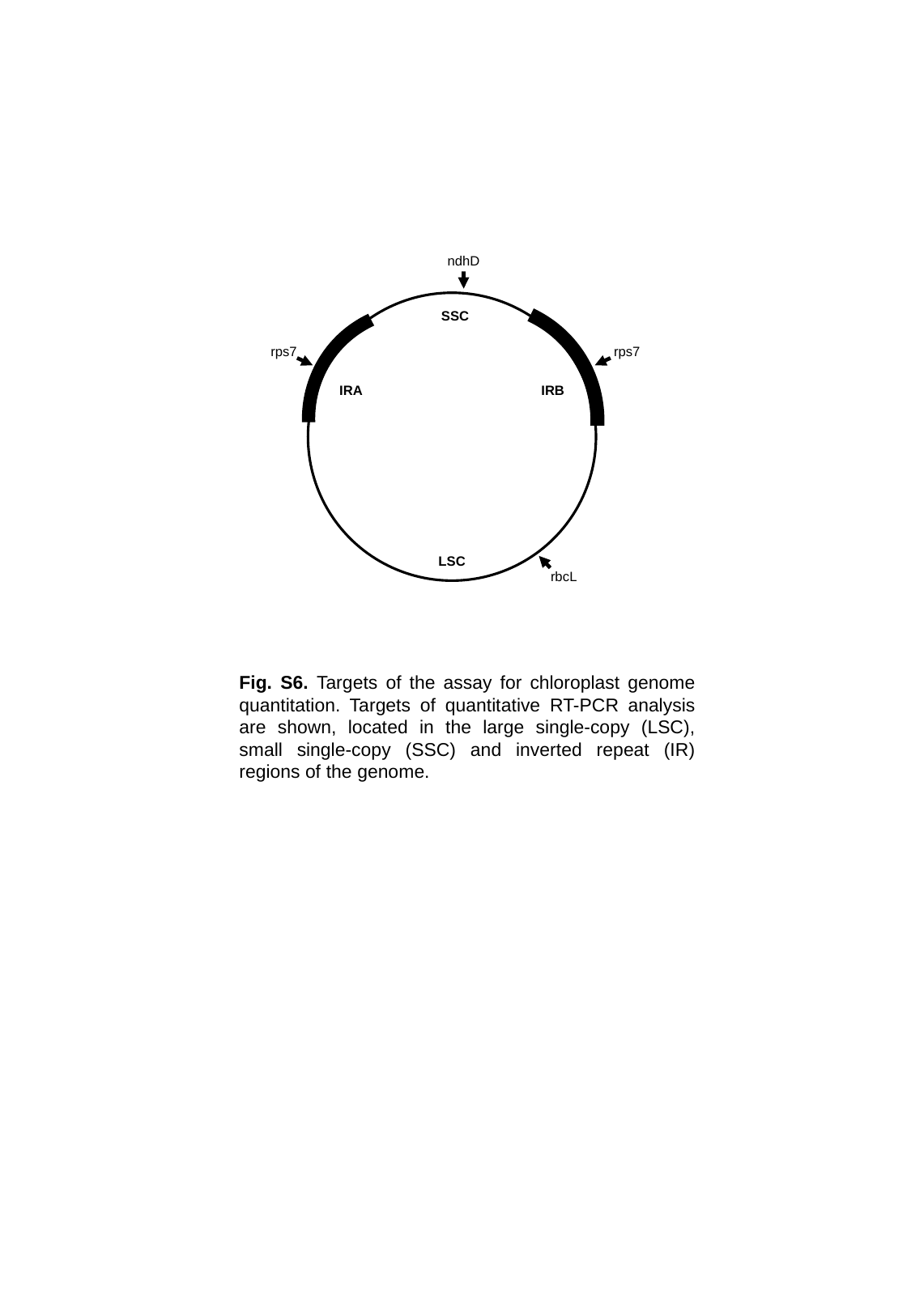

ndhD
SSC
IRA
IRB
LSC
rps7
rps7
rbcL
Fig. S6. Targets of the assay for chloroplast genome quantitation. Targets of quantitative RT-PCR analysis are shown, located in the large single-copy (LSC), small single-copy (SSC) and inverted repeat (IR) regions of the genome.

## Slide 7
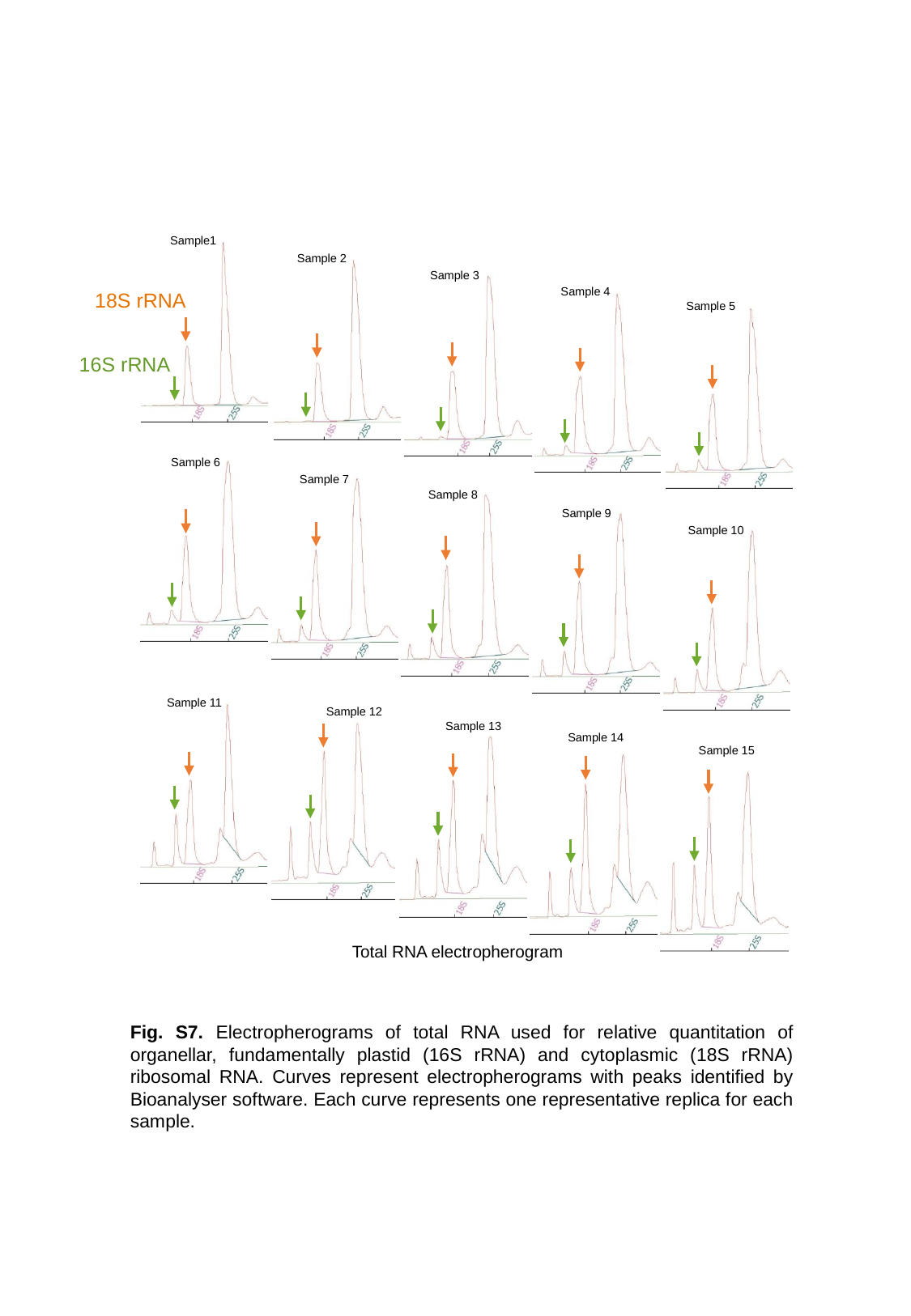

Sample1
Sample 2
Sample 3
Sample 4
18S rRNA
Sample 5
16S rRNA
Sample 6
Sample 7
Sample 8
Sample 9
Sample 10
Sample 11
Sample 12
Sample 13
Sample 14
Sample 15
Total RNA electropherogram
Fig. S7. Electropherograms of total RNA used for relative quantitation of organellar, fundamentally plastid (16S rRNA) and cytoplasmic (18S rRNA) ribosomal RNA. Curves represent electropherograms with peaks identified by Bioanalyser software. Each curve represents one representative replica for each sample.

## Slide 8
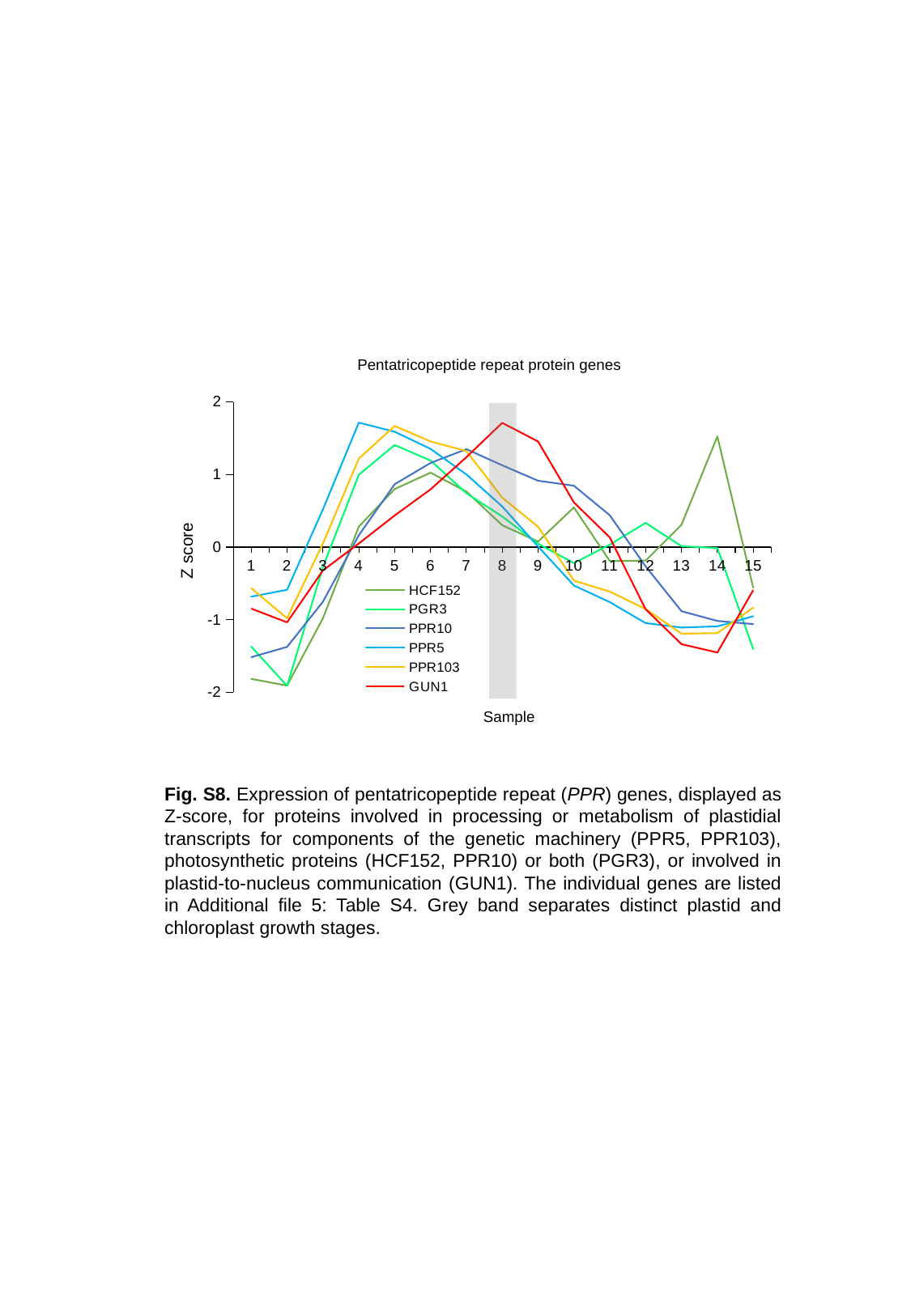

### Chart
| Category | HCF152 | PGR3 | PPR10 | PPR5 | PPR103 | GUN1 |
|---|---|---|---|---|---|---|Pentatricopeptide repeat protein genes
Z score
Sample
Fig. S8. Expression of pentatricopeptide repeat (PPR) genes, displayed as Z-score, for proteins involved in processing or metabolism of plastidial transcripts for components of the genetic machinery (PPR5, PPR103), photosynthetic proteins (HCF152, PPR10) or both (PGR3), or involved in plastid-to-nucleus communication (GUN1). The individual genes are listed in Additional file 5: Table S4. Grey band separates distinct plastid and chloroplast growth stages.

## Slide 9
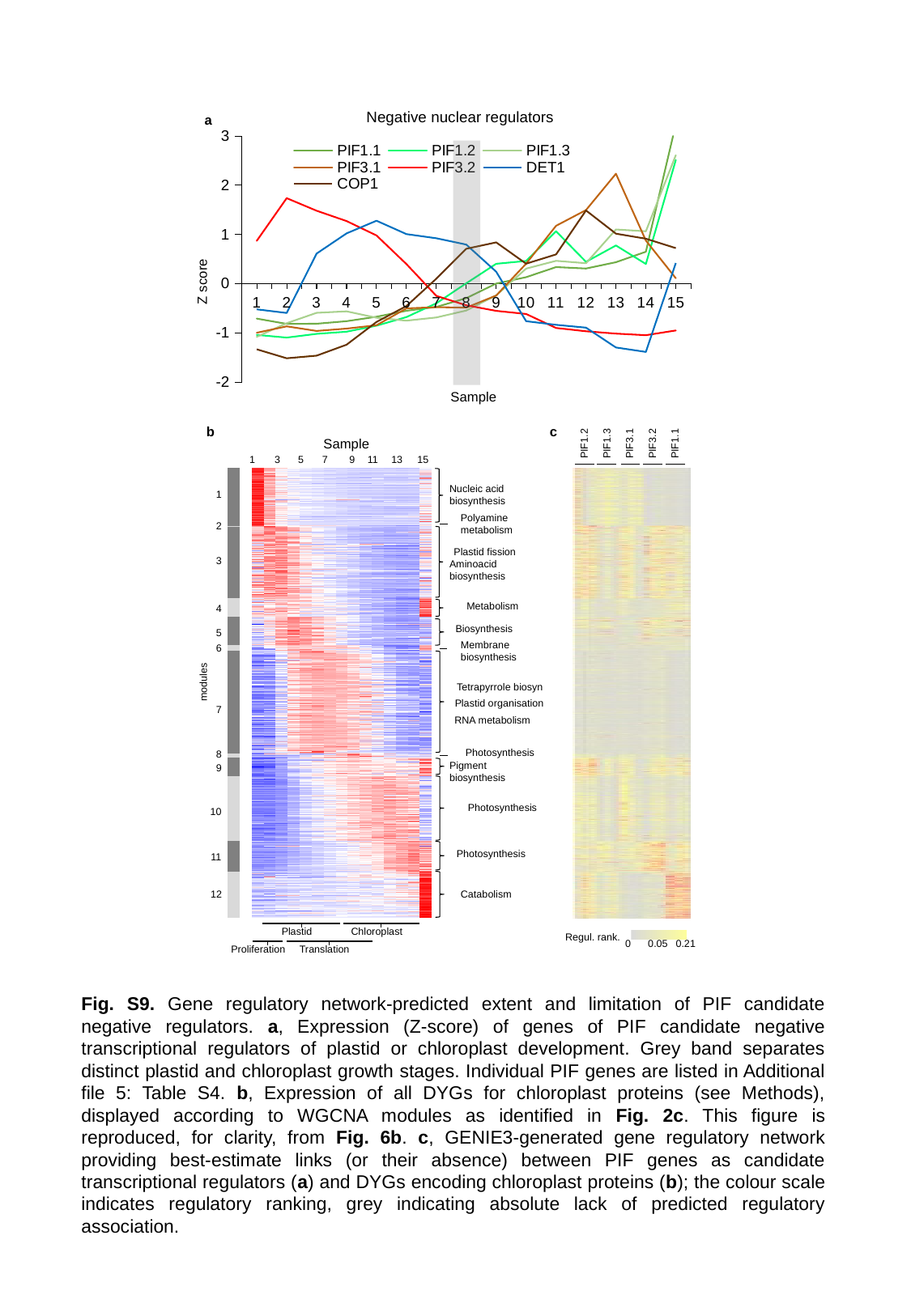

### Chart
| Category | PIF1.1 | PIF1.2 | PIF1.3 | PIF3.1 | PIF3.2 | DET1 | COP1 |
|---|---|---|---|---|---|---|---|
Z score
Sample
Negative nuclear regulators
a
c
PIF1.2
PIF1.3
PIF3.1
PIF3.2
PIF1.1
Regul. rank.
0
0.05
0.21
b
Sample
1
3
5
7
9
11
13
15
Nucleic acid biosynthesis
1
Polyamine metabolism
2
Plastid fission
3
Aminoacid biosynthesis
Metabolism
4
Biosynthesis
5
Membrane biosynthesis
6
modules
Tetrapyrrole biosyn
Plastid organisation
7
RNA metabolism
Photosynthesis
8
Pigment biosynthesis
9
Photosynthesis
10
Photosynthesis
11
Catabolism
12
Plastid
Chloroplast
Proliferation
Translation
Fig. S9. Gene regulatory network-predicted extent and limitation of PIF candidate negative regulators. a, Expression (Z-score) of genes of PIF candidate negative transcriptional regulators of plastid or chloroplast development. Grey band separates distinct plastid and chloroplast growth stages. Individual PIF genes are listed in Additional file 5: Table S4. b, Expression of all DYGs for chloroplast proteins (see Methods), displayed according to WGCNA modules as identified in Fig. 2c. This figure is reproduced, for clarity, from Fig. 6b. c, GENIE3-generated gene regulatory network providing best-estimate links (or their absence) between PIF genes as candidate transcriptional regulators (a) and DYGs encoding chloroplast proteins (b); the colour scale indicates regulatory ranking, grey indicating absolute lack of predicted regulatory association.
