# Supplementary material for: Cellular and transcriptomic analyses reveal two-staged chloroplast biogenesis underpinning photosynthesis build-up in the wheat leaf
Source: Genome Biol. 2021 May 11;22:151. doi: 10.1186/s13059-021-02366-3 (PMC8111775; doi:10.1186/s13059-021-02366-3)
Supplement: Supplementary file 5 — Additional file 5: Table S4. List of genes representative of key biological functions whose expression was individually plotted in Figs. 3, 4, 5, 6 and Additional file 1 Figures 8-9. Table S5. Primers used for genome copy number and rRNA quantitation. Table S6. Summary of quantitative cellular and other parameters and data analysis. Table S7. R script used to generate box plots. Table S8. Correction parameters for cell age and mitosis. [file 13059_2021_2366_MOESM5_ESM.docx]

**Table S4. List of genes representative of key biological functions whose expression was individually plotted in Figs. 3 to 6 and Additional file 1 Figs. S8 and S9**

**Cell cycle**

| **Gene ID** | **Name** | **Function** |  | **Gene ID** | **Name** | **Function** |
| --- | --- | --- | --- | --- | --- | --- |
| TraesCS1B02G320900 | CYCB1.2 | S-phase |  | TraesCS2A02G214700 | HTA2 | S-phase |
| TraesCS1D02G309400 |  |  |  | TraesCS2B02G239600 |  |  |
| TraesCS3A02G333000 |  |  |  | TraesCS2B02G239700 |  |  |
| TraesCS3B02G363200 |  |  |  | TraesCS2D02G220400 |  |  |
| TraesCS3D02G195200 |  |  |  | TraesCS2D02G220500 |  |  |
| TraesCS3D02G326600 |  |  |  | TraesCS2A02G364000 | PCNA2 |  |
| TraesCS4A02G143200 | RBR1 |  |  | TraesCS2B02G382000 |  |  |
| TraesCS4B02G162200 |  |  |  | TraesCS2D02G361800 |  |  |
| TraesCS4D02G159600 |  |  |  | TraesCS4B02G366100 |  |  |
| TraesCS7A02G264800 | RBR2 |  |  | TraesCS6A02G373400 |  |  |
| TraesCS7B02G162700 |  |  |  | TraesCS6B02G411100 |  |  |
| TraesCS7D02G265600 |  |  |  | TraesCS6D02G357600 |  |  |
| TraesCS6A02G373000 | RNR1 |  |  | TraesCS4A02G192300 | CDKB1.2 | Mitosis |
| TraesCS6B02G410800 |  |  |  | TraesCS4B02G123100 |  |  |
| TraesCS6D02G357100 |  |  |  | TraesCS4D02G121200 |  |  |
| TraesCS7A02G131200 |  |  |  | TraesCS5A02G145500 |  |  |
| TraesCS7B02G031500 |  |  |  | TraesCS5B02G144100 |  |  |
| TraesCS7D02G130500 |  |  |  | TraesCS5D02G147100 |  |  |

**Cellular growth**

| **Gene ID** | **Name** | **Function** |  | **Gene ID** | **Name** | **Function** |
| --- | --- | --- | --- | --- | --- | --- |
| TraesCS2A02G065700 | PIP3B | Aquaporins |  | TraesCS1A02G116200 | CESA1 | Cellulose synthesis |
| TraesCS2B02G077700 |  |  |  | TraesCS1B02G136200 |  |  |
| TraesCS2D02G063900 |  |  |  | TraesCS1D02G117400 |  |  |
| TraesCS6B02G259000 | PIP2_1 |  |  | TraesCS6A02G077800 |  |  |
| TraesCS6D02G212900 |  |  |  | TraesCS6B02G104600 |  |  |
| TraesCS6A02G222100 |  |  |  | TraesCSU02G142500 |  |  |
| TraesCS2B02G396700 |  |  |  | TraesCS2A02G157100 | CSLA9_1 |  |
| TraesCS2D02G376000 |  |  |  | TraesCS2B02G182700 |  |  |
| TraesCS2A02G379800 |  |  |  | TraesCS2D02G163000 |  |  |
| TraesCS2A02G379900 |  |  |  | TraesCS3A02G496400 |  |  |
| TraesCS2B02G396800 |  |  |  | TraesCS3D02G504200 |  |  |
| TraesCS2D02G376100 |  |  |  | TraesCS3B02G558400 |  |  |
| TraesCS7A02G343600 | AGP16_1 | Arabinogalactan  proteins |  | TraesCS7A02G189100 | CSLA9_3 |  |
| TraesCS7B02G239500 |  |  |  | TraesCS7D02G190000 |  |  |
| TraesCS7D02G336000 |  |  |  | TraesCS5B02G517500 | CESA6 |  |
| TraesCS1A02G113100 |  |  |  | TraesCS5D02G517200 |  |  |
| TraesCS1B02G133200 |  |  |  | TraesCS4A02G355000 |  |  |
| TraesCS1D02G114500 |  |  |  |  |  |  |
| TraesCS5A02G481200 | EXPA4 | Expansins |  |  |  |  |
| TraesCS5B02G494400 |  |  |  |  |  |  |
| TraesCS2A02G393700 | EXP13_1 |  |  |  |  |  |
| TraesCS2D02G391600 |  |  |  |  |  |  |
| TraesCS2B02G411700 | EXP13_2 |  |  |  |  |  |

**Chloroplast division**

| **Gene ID** | **Name** |  | **Gene ID** | **Name** |
| --- | --- | --- | --- | --- |
| TraesCS3D02G157200 | PDV1 |  | TraesCS4A02G315500 | ARC2 |
| TraesCS2D02G257600 | PDV2 |  | TraesCS5B02G563900 |  |
| TraesCS2A02G266200 |  |  | TraesCS5D02G550700 |  |
| TraesCS2B02G275900 |  |  | TraesCS5B02G120600 | ARC5 |
| TraesCS2A02G488400 | FtsZ1 |  | TraesCS5D02G124800 |  |
| TraesCS2B02G516000 |  |  | TraesCS5A02G114600 |  |
| TraesCS2D02G488700 |  |  | TraesCS6A02G066200 | ARC6 |
| TraesCS1A02G276500 | FtsZ2-1 |  | TraesCS6B02G089500 |  |
| TraesCS1B02G285700 |  |  | TraesCSU02G117700 |  |
| TraesCS1D02G276000 |  |  | TraesCS1A02G243700 | FZL |
| TraesCS2A02G294500 |  |  | TraesCS1B02G255100 |  |
| TraesCS2B02G310900 |  |  | TraesCS1D02G243700 |  |
| TraesCS2D02G292200 |  |  |  |  |

**Chloroplast protein import machinery**

| **Gene ID** | **Name** |  | **Gene ID** | **Name** |
| --- | --- | --- | --- | --- |
| TraesCS4A02G095300 | TOC75-III |  | TraesCS2A02G199800 | TIC20-I |
| TraesCS4B02G209200 |  |  | TraesCS2B02G227100 |  |
| TraesCS4D02G209900 |  |  | TraesCS2D02G207600 |  |
| TraesCS4A02G072800 | TOC34 |  | TraesCS6A02G229400 |  |
| TraesCS4B02G228900 |  |  | TraesCS6B02G252100 |  |
| TraesCS4D02G230000 |  |  | TraesCS6D02G205400 |  |
| TraesCS1A02G208000 | TOC132 |  | TraesCS1A02G165800 | TIC110 |
| TraesCS1B02G222000 |  |  | TraesCS1B02G182500 |  |
| TraesCS1D02G211300 |  |  | TraesCS1D02G174900 |  |
| TraesCS1A02G093900 | TOC159_1 |  | TraesCS1A02G165700 |  |
| TraesCS1B02G122000 |  |  | TraesCS1B02G182400 |  |
| TraesCS1D02G102500 |  |  | TraesCS1D02G175000 |  |
| TraesCS5B02G514000 | TOC159_2 |  | TraesCS5A02G206800 | HSP21 |
| TraesCS5D02G514500 |  |  | TraesCS5B02G205200 |  |
| TraesCS4A02G358400 |  |  | TraesCS5D02G213000 |  |
| TraesCS2A02G138100 | SP1 |  | TraesCS5A02G206900 |  |
| TraesCS2B02G162000 |  |  | TraesCS5A02G257700 |  |
| TraesCS2D02G141100 |  |  | TraesCS5B02G205300 |  |
| TraesCS6A02G158800 | OEP80 |  | TraesCS5B02G257000 |  |
| TraesCS6D02G153700 |  |  | TraesCS5D02G213100 |  |
| TraesCS6B02G192500 |  |  | TraesCS1A02G133100 | cpHSC70-2_1 |
| TraesCS2D02G306800 | TIC40 |  | TraesCS1B02G151300 |  |
| TraesCS2A02G308600 |  |  | TraesCS1D02G131800 |  |
| TraesCS2B02G325100 |  |  | TraesCS5A02G106200 | cpHSC70-2_2 |
|  |  |  | TraesCS5B02G111200 |  |
|  |  |  | TraesCS5D02G117600 |  |

**Chloroplast nucleoid-associated proteins**

| **Gene ID** | **Name** |  | **Gene ID** | **Name** |
| --- | --- | --- | --- | --- |
| TraesCS5A02G486300 | PTAC2 |  | TraesCS3A02G365700 | FLN1 |
| TraesCS5B02G500100 |  |  | TraesCS3B02G397500 |  |
| TraesCS5D02G500300 |  |  | TraesCS3D02G358800 |  |
| TraesCS3B02G425900 | PTAC14 |  | TraesCS1B02G222400 | MurE-like |
| TraesCS3D02G387600 |  |  | TraesCS1D02G211600 |  |
| TraesCS3A02G393900 |  |  | TraesCS1A02G208400 |  |
| TraesCS1A02G427200 | PTAC10 |  | TraesCS4A02G395700 | WHY3 / PTAC11 |
| TraesCS1B02G462200 |  |  | TraesCS7A02G095300 |  |
| TraesCS1D02G437000 |  |  | TraesCS7D02G091500 |  |
| TraesCS3A02G297500 | PTAC12 / HEMERA |  |  | |
| TraesCS3B02G339200 |  |  |  |  |
| TraesCS3D02G304800 |  |  |  |  |

**Chloroplast transcription**

| **Gene ID** | **Name** |  | **Gene ID** | **Name** |
| --- | --- | --- | --- | --- |
| TraesCS7A02G503900 | RPOTp / SCABRA3 |  | TraesCS1A02G431600 | SIG3 |
| TraesCS7B02G411000 |  |  | TraesCS1B02G467100 |  |
| TraesCS7A02G297900 | SIG1 |  | TraesCS1D02G440900 |  |
| TraesCS7B02G184800 |  |  | TraesCS1A02G426700 | SIG5 |
| TraesCS7D02G292000 |  |  | TraesCS1B02G461600 |  |
| TraesCS4A02G157100 | SIG2_1 |  | TraesCS1D02G436500 |  |
| TraesCS4B02G163500 |  |  | TraesCS5B02G563800 | SIG6 |
| TraesCS4D02G154500 |  |  | TraesCS5D02G551200 |  |
| TraesCS4A02G095200 | SIG2_2 |  | TraesCS4A02G315700 |  |
| TraesCS4B02G209300 |  |  | TraesCS3D02G342900 | RCB / SVR4 |
| TraesCS4D02G210000 |  |  | TraesCS3B02G381100 |  |
|  |  |  | TraesCS3A02G349000 |  |

**Chloroplast translation**

| **Gene ID** | **Name** |  | **Gene ID** | **Name** |
| --- | --- | --- | --- | --- |
| TraesCS4B02G347000 | PRPL11 |  | TraesCS3A02G275700 | W tRNA-Syn |
| TraesCS4D02G341900 |  |  | TraesCS3B02G309400 |  |
| TraesCS5A02G515700 |  |  | TraesCS3D02G275600 |  |
| TraesCS5A02G404500 | PRPS9 |  | TraesCS4A02G079200 | SVR7 |
| TraesCS5B02G409400 |  |  | TraesCS4B02G245000 |  |
| TraesCS5D02G414600 |  |  | TraesCS4D02G244400 |  |
| TraesCS6A02G319100 | PRPL29 |  | TraesCS6A02G209800 | EF-TU / SVR11 |
| TraesCS6B02G349300 |  |  | TraesCS6B02G238600 |  |
| TraesCS6D02G298300 |  |  | TraesCS6D02G193000 |  |
| TraesCS2A02G387000 | SCO1 |  | TraesCS7D02G288700 | FUG1 |
| TraesCS2B02G404600 |  |  | TraesCS7A02G287900 |  |
| TraesCS2D02G383800 |  |  | TraesCS7B02G183700 |  |

**Chloroplast coverage**

| **Gene ID** | **Name** |
| --- | --- |
| TraesCS3A02G303900 | REC1 |
| TraesCS3A02G304100 |  |
| TraesCS3B02G332300 |  |
| TraesCS3B02G332400 |  |
| TraesCS3D02G297800 |  |
| TraesCS3D02G297900 |  |
| TraesCS2A02G517100 | REC2 |
| TraesCS6A02G354400 |  |
| TraesCS6B02G387400 |  |
| TraesCS6D02G336700 |  |
| TraesCS6D02G336800 |  |
| TraesCS2B02G545600 | REC3 |
| TraesCS2D02G518700 |  |
| TraesCS6A02G280800 | FMT |
| TraesCS6D02G261100 |  |
| TraesCS6B02G309600 |  |

**Pigment / Thylakoid development**

| **Gene ID** | **Name** | **Function** |
| --- | --- | --- |
| TraesCS1A02G057200 | HEMA1_1 | Chlorophyll synthesis |
| TraesCS1A02G173100 |  |  |
| TraesCS1B02G075200 |  |  |
| TraesCS1B02G191200 |  |  |
| TraesCS1D02G058300 |  |  |
| TraesCS1D02G165600 |  |  |
| TraesCS2A02G134000 | CHLH/ GUN5 |  |
| TraesCS2B02G157600 |  |  |
| TraesCS2D02G136200 |  |  |
| TraesCS3A02G506200 | CAO |  |
| TraesCS3B02G574300 |  |  |
| TraesCS3D02G514100 |  |  |
| TraesCS5A02G020900 | PSY | Carotenoid synthesis |
| TraesCS5D02G026000 |  |  |
| TraesCS7A02G557300 |  |  |
| TraesCS7B02G482000 |  |  |
| TraesCS7D02G553300 |  |  |
| TraesCS5B02G017900 |  |  |
| TraesCS4A02G004900 | PDS3 |  |
| TraesCS4B02G300100 |  |  |
| TraesCS4D02G299000 |  |  |
| TraesCS3A02G304900 | CURT1 | Thylakoid development |
| TraesCS3B02G331600 |  |  |
| TraesCS3D02G296900 |  |  |

**Photophosphorylation, Calvin cycle and photorespiration**

| **Gene ID** | **Name** | **Function** |
| --- | --- | --- |
| TraesCS2A02G247300 | PSBO2 | Reaction centres |
| TraesCS2B02G270300 |  |  |
| TraesCS2D02G248400 |  |  |
| TraesCS5A02G482800 | PSAE2 |  |
| TraesCS5B02G496000 |  |  |
| TraesCS5D02G496400 |  |  |
| TraesCS7A02G227100 | LHCA1 | Antenna |
| TraesCS7B02G192500 |  |  |
| TraesCS7D02G227300 |  |  |
| TraesCS5B02G463000 | LHCB1.3 |  |
| TraesCS5B02G462900 |  |  |
| TraesCS5B02G462800 |  |  |
| TraesCS7A02G276400 | LHCB1.4 |  |
| TraesCS7D02G276300 |  |  |
| TraesCS1B02G317500 |  |  |
| TraesCSU02G086800 |  |  |
| TraesCS4A02G499500 | PETE1 | Electron transport |
| TraesCS7A02G000400 |  |  |
| TraesCS7D02G000300 |  |  |
| TraesCS2A02G223600 | ATPC1 | ATP synthase |
| TraesCS2B02G273900 |  |  |
| TraesCS2D02G229600 |  |  |
| TraesCS2A02G066700 | RBCS | Calvin cycle |
| TraesCS2B02G078900 |  |  |
| TraesCS2D02G065100 |  |  |
| TraesCS3A02G367000 | SBPASE |  |
| TraesCS3B02G398300 |  |  |
| TraesCS3D02G359900 |  |  |
| TraesCS2D02G346900 | PGLP1 |  |
| TraesCS2B02G366900 |  |  |
| TraesCS2A02G348500 |  |  |
| TraesCS3D02G266100 | GLYK | Photorespiration |
| TraesCS3B02G299300 |  |  |
| TraesCS3A02G265900 |  |  |

**Transcripts and immunoblotted proteins representative of two stages of plastid development**

| **Gene ID** | **Name** | **Stage** |
| --- | --- | --- |
| TraesCS3D02G342900 | RCB/ SVR4 | Plastid |
| TraesCS3B02G381100 |  |  |
| TraesCS3A02G349000 |  |  |
| TraesCS2D02G306800 | TIC40 |  |
| TraesCS2A02G308600 |  |  |
| TraesCS2B02G325100 |  |  |
| TraesCS5B02G120600 | ARC5 |  |
| TraesCS5D02G124800 |  |  |
| TraesCS5A02G114600 |  |  |
| TraesCS2A02G247300 | PSBO2 | Chloroplast |
| TraesCS2B02G270300 |  |  |
| TraesCS2D02G248400 |  |  |
| TraesCS7A02G276400 | LHCB1.4 |  |
| TraesCS7D02G276300 |  |  |
| TraesCS1B02G317500 |  |  |
| TraesCSU02G086800 |  |  |
| TraesCS3A02G367000 | SBPASE |  |
| TraesCS3B02G398300 |  |  |
| TraesCS3D02G359900 |  |  |

**Nuclear regulators of plastid or chloroplast development**

| **Gene ID** | **Name** | **Function** |
| --- | --- | --- |
| TraesCS7A02G422200 | CIA2_1 | Protein import |
| TraesCS7B02G322600 |  |  |
| TraesCS7D02G414400 |  |  |
| TraesCS6A02G118000 | CIA2_2 |  |
| TraesCS6B02G146200 |  |  |
| TraesCS6D02G108000 |  |  |
| TraesCS7A02G339800 | GLK1_1 | Pigment synthesis |
| TraesCS7B02G251400 |  |  |
| TraesCS7D02G347500 |  |  |
| TraesCS3A02G161000 | GLK1_2 |  |
| TraesCS3B02G191600 |  |  |
| TraesCS3D02G168200 |  |  |
| TraesCS3A02G128900 | HY5_1 | Photomorphogenesis |
| TraesCS3D02G129800 |  |  |
| TraesCS6A02G175800 | HY5_2 |  |
| TraesCS6B02G209600 |  |  |
| TraesCS6D02G167800 |  |  |
| TraesCS3A02G297500 | PTAC12 / HEMERA | Plastid biogenesis,  photomorphogenesis |
| TraesCS3B02G339200 |  |  |
| TraesCS3D02G304800 |  |  |
| TraesCS1B02G270800 | NCP |  |
| TraesCS1D02G260200 |  |  |
| TraesCS1A02G260300 |  |  |
| TraesCS7A02G363100 | GNC | Cytokinin signaling |
| TraesCS7B02G264800 |  |  |
| TraesCS7D02G359900 |  |  |
| TraesCS2A02G253900 | PIF1.1 | Suppression of photomor-phogenesis (transcriptional) |
| TraesCS2B02G273500 |  |  |
| TraesCS2D02G254400 |  |  |
| TraesCS5A02G420200 | PIF1.2 |  |
| TraesCS5B02G422000 |  |  |
| TraesCS5D02G428400 |  |  |
| TraesCS5D02G386500 | PIF1.3 |  |
| TraesCS5A02G376500 |  |  |
| TraesCS5B02G380200 |  |  |
| TraesCS5A02G049600 | PIF3.1 |  |
| TraesCS5B02G054800 |  |  |
| TraesCS5D02G060300 |  |  |
| TraesCS1A02G083000 | PIF3.2 |  |
| TraesCS1B02G100400 |  |  |
| TraesCS1D02G084200 |  |  |
| TraesCS6A02G326100 | COP1 | Suppression of photomor-phogenesis (via protein turnover) |
| TraesCS6B02G356400 |  |  |
| TraesCS6D02G305800 |  |  |
| TraesCS3B02G231100 | DET1 |  |
| TraesCS3A02G194600 |  |  |
| TraesCS4A02G125100 |  |  |
| TraesCS4D02G180900 |  |  |

**Pentatricopeptide repeat (PPR) proteins**

| **Gene ID** | **Name** | **Function** |
| --- | --- | --- |
| TraesCS5A02G085800 | GUN1 | Retrograde signalling |
| TraesCS5D02G097800 |  |  |
| TraesCS5B02G091600 |  |  |
| TraesCS5D02G160900 | HCF152 | Processing transcripts of housekeeping proteins |
| TraesCS5A02G155800 |  |  |
| TraesCS7A02G495500 | PPR10 |  |
| TraesCS7B02G401800 |  |  |
| TraesCS7D02G482800 |  |  |
| TraesCS6A02G303300 | PPR5 | Processing transcripts of photosynthesis proteins |
| TraesCS6B02G332800 |  |  |
| TraesCS6D02G282900 |  |  |
| TraesCS2A02G403371 | PPR103 |  |
| TraesCS2B02G421400 |  |  |
| TraesCS2D02G400500 |  |  |
| TraesCS4A02G246600 | PGR3 | Processing transcripts of both housekeeping and photosynthesis proteins |
| TraesCS4D02G067300 |  |  |
| TraesCS4B02G068200 |  |  |

**Table S5. Primers used for genome copy number and rRNA quantitation**

**Primers used for chloroplast genome copy number**

| **Region** | **Gene ID** | **Forward / Reverse** | **Primer sequence** |
| --- | --- | --- | --- |
| Nucleus | TraesCS2A02G429600  TraesCS2B02G450100  TraesCS2D02G427200 | Ta_KO_F | TGCTACATGTGACTATGGTGAC |
|  |  | Ta_KO_R | AGCAGAAGAACCCAACAAACC |
|  | TraesCS2A02G425400  TraesCS2B02G445700  TraesCS2D02G423300 | Ta_KS_F | CAAGGAGCTGTTCTGGAAGA |
|  |  | Ta_KS_R | TCGTTGATAACAGCATTCACCG |
| Chloroplast  (Large single copy) | NC_002762.1 gene ID: 803091 | Ta_rbcL_F | AGCAGGTGTTGGATTTAAAGCTG |
|  |  | Ta_rbcL_R | ACTCGGAATGCTGCCAAGAT |
| Chloroplast  (Small single copy) | NC_002762.1 gene ID: 803119 | Ta_ndhD_F | GTTCTCGTGGTCCAGAATCCA |
|  |  | Ta_ndhD_R | TGTTACGCCAGATGTTCTATGGA |
| Chloroplast  (Inverted repeat) | NC_002762.1 gene ID: 803210 and 803088 | Ta_rps7_F | TCTGCCATTCTATGAGTCGCT |
|  |  | Ta_rps7_R | TCCGAATTAGTAGATGCTGCCA |

**Primers used for the reverse transcription (RT), and RT qPCR of 18S rRNA and 16S rRNA**

| **Region** | **Oligo Name** | **Function** | **Sequence** |
| --- | --- | --- | --- |
| 18S rRNA and 16S rRNA | Ta_rRNA_RT_F | RT | ACCTTGTTACGACTTC |
| 18S rRNA | Ta_18SrRNA _F | qPCR | TGAAAGACGAACAACTGCGAAAG |
|  | Ta_18SrRNA _R | qPCR | TGGTTGAGACTAGGACGGTATCT |
| 16S rRNA | Ta_16SrRNA _F | qPCR | CATCGGCTAACTCTGTGCCA |
|  | Ta_16SrRNA _R | qPCR | GGTTGAGCCCTGGGATTTGA |

**Table S6. Summary of quantitative cellular and other parameters and data analysis**

| **Parameter** | **Calculation** | **Significance** |
| --- | --- | --- |
| - | For microscopic data, 4 cells of each of 4 cell preparations of each of 12 independent leaves were measured (n=48 cells) for each numbered section | - |
| Cell area | Mean cell plan area (n=48) for each section | Expansion of mesophyll cells |
| Chloroplast area | Mean chloroplast area (n=10 in each cell) from 48 cells per section | Growth of individual chloroplasts along the developing leaf |
| Chloroplast Count | Mean of total number of chloroplasts per cell in 48 cells | Chloroplast number per cell as a result of plastid proliferation |
| Plastid division rate | Change in plastid count between each sample and the subsequent one, X correction factor for the rate of cell division indicated in Table S8 | Estimated rate of plastid proliferation |
| Total chloroplast area | Total chloroplast number per cell X mean chloroplast area of the same cell, calculated per cell, mean for 48 cells | Total chloroplast cellular build-up |
| Cellular chloroplast growth rate | Change of the total plan area of the total chloroplast population of one cell, between each sample and the subsequent one, X correction factor for the effect of cell division indicated in Table S8 | Rate of build-up of total chloroplast cellular content |
| Chloroplast compartment | (Total chlp. number X mean chlp. area) / cell area, calculated per cell, mean for 48 cells. | Proportion of the cell plan area occupied by chloroplasts |
| - | DNA and RNA extracted from 4 biological replicates with 12 independent leaves per replicate for each numbered section | - |
| Chloroplast genome copy number per cell’s haploid genome | cpDNA / gDNA, absolute quantitation (3 replicates used) | Chloroplast DNA replication; investment into chloroplast genomes |
| Chloroplast ribosome build-up | 16S rRNA / 18S rRNA peak areas | Investement in chloroplast translation capacity |
| Chlorophyll content | Chl.Tot. = 19.43*A_646.8_ + 8.05*A_663.8_  2 seedlings per replicate, 3 biological replicates | Photosynthetic apparatus establishement along the developing leaf |

**Table S7. R script used to generate box plots**

| Colour gradient | - colour<-colorRampPalette(c("white", "yellow", "dark green")) - colour(15) |
| --- | --- |
| Cell area | - boxplot(Cell_area$Cell_area~Cell_area$Sample, outline=FALSE, col=colour(15), names=c("1", "2", "3", "4", "5", "6", "7", "8", "9", "10", "11", "12", "13", "14", "15"), las=1, boxwex=0.5, cex.axis=1.1) |
| Chloroplast area | - boxplot(Mean_chloroplastarea$Mean_chloroplast_area~Mean_chloroplastarea$Sample, outline=FALSE, col=colour(15), names=c("1", "2", "3", "4", "5", "6", "7", "8", "9", "10", "11", "12", "13", "14", "15"), las=1, boxwex=0.5, cex.axis=1.1) |
| Chloroplast count | - boxplot(Chloroplast_count$Chloroplast_count~Chloroplast_count$Sample, outline=FALSE, col=colour(15), names=c("1", "2", "3", "4", "5", "6", "7", "8", "9", "10", "11", "12", "13", "14", "15"), las=1, boxwex=0.5, cex.axis=1.1) |
| Total chloroplast area | - boxplot(Total_chloroplastarea$Total_chloroplast_area~Total_chloroplastarea$Sample, outline=FALSE, col=colour(15), names=c("1", "2", "3", "4", "5", "6", "7", "8", "9", "10", "11", "12", "13", "14", "15"), las=1, boxwex=0.5, cex.axis=1.1) |
| Chloroplast compartment | - boxplot(Chloroplast_compartment$Chloroplast_compartment~Chloroplast_compartment$Sample, outline=FALSE, col=colour(15), names=c("1", "2", "3", "4", "5", "6", "7", "8", "9", "10", "11", "12", "13", "14", "15"), las=1, boxwex=0.5, cex.axis=1.1, ylim=c(0,200)) |

**Table S8. Correction parameters for cell age and mitosis**

| **Sample No.** | **Section** | **Mid point (mm)** | **Cell age (h) estimated from Boffey 1980, corrected for observed elongation rate^†^** | **Nuclei in S phase (%)*** | **Correction factor for cell division^†^** |
| --- | --- | --- | --- | --- | --- |
| 1 | M+L3 (~3mm) | 0 | 0 | 29.8 | 2.00 |
| 2 | 0-5mm | 2.5 | 16.5 | 26.5 | 1.89 |
| 3 | 5-10mm | 7.5 | 23 | 10.2 | 1.31 |
| 4 | 10-15mm | 12.5 | 27 | 4.2 | 1.10 |
| 5 | 15-20mm | 17.5 | 31 | 2.2 | 1.03 |
| 6 | 20-25mm | 22.5 | 35 | 3.4 | 1.07 |
| 7 | 25-30mm | 27.5 | 37.5 | 2.7 | 1.05 |
| 8 | 30-35mm | 32.5 | 40 | 2.9 | 1.06 |
| 9 | 35-40mm | 37.5 | 43 | 2.8 | 1.05 |
| 10 | 40-50mm | 45 | 48 | 2.5 | 1.04 |
| 11 | 50-60mm | 55 | 55 | 1.6 | 1.01 |
| 12 | 70-80mm | 75 | 66 | 1.1 | 0.99 |
| 13 | 100-110mm | 105 | 83 | 1.5 | 1.01 |
| 14 | 130-140mm | 135 | 100 | 1.0 | 0.99 |
| 15 | 180-200mm | 190 | 290 | - | - |

*Measured value

^†^Estimate or correction factor
