# Supplementary material for: Cellular and transcriptomic analyses reveal two-staged chloroplast biogenesis underpinning photosynthesis build-up in the wheat leaf
Source: Genome Biol. 2021 May 11;22:151. doi: 10.1186/s13059-021-02366-3 (PMC8111775; doi:10.1186/s13059-021-02366-3)
Supplement: Supplementary file 8 — Additional file 8. Review history. [file 13059_2021_2366_MOESM8_ESM.docx]

Review History

**First round of review**

**Reviewer 1**

**Are you able to assess all statistics in the manuscript, including the appropriateness of statistical tests used?**

No, I do not feel adequately qualified to assess the statistics.

**Comments to author:**

This study takes advantage of a powerful and under-appreciated system - the developmental progression of photosynthetic differentiation along the leaf blade of grasses - to gain insight into the regulatory networks underlying chloroplast biogenesis. The experiments use wheat, whose leaf sections are characterized by RNA-seq, microscopy, DNA content, and more. The boundaries of the leaf sections were thoughtfully selected based on an estimate of cellular age, such that sampling was denser near the leaf base than the tip. Although prior studies have characterized transcriptomes, proteomes, and other properties along the leaf blade of grasses, no prior study has integrated transcriptome data with features such as chloroplast division and DNA replication, or discussed transcriptome dynamics in the context of chloroplast biogenesis. There is a wealth of information in this paper, and it will surely be of interest to the field. However, its impact would be greater if certain points were clarified and if prior work in this area were discussed in the context of the new data. For example:

i. Prior studies addressing related issues using cereal leaf blades are cited in the Introduction (references 7-14), but there is no attempt to integrate the findings from those studies with the results presented here. Furthermore, some seminal studies of this type are not cited at all. These include:

- classic work on chloroplast development along the barley leaf blade from the Mullet lab, which address things like the developmental profile of buildup of the chloroplast genome, translation machinery, etc.

http://www.plantphysiol.org/content/89/3/1011.long

http://www.plantphysiol.org/content/plantphysiol/101/3/781.full.pdf

- a comparative analysis of transcriptome dynamics along maize and rice seedling leaf blades (Brutnell lab) https://www.nature.com/articles/nbt.3019

It is important to discuss the body of prior work on this topic (both the cited papers, and those that should be cited) in the context of the new results here.

ii) It would be helpful to provide a brief explanation of (i) how the different "components" resulting from PCA are determined, what they signify and the interpretation of Figure 2a; (ii) the term "gene load factor"; (iii) what is shown on the X and Y axis in Fig S4.

iii) Fig. 2 and Fig S2. The functional terms "ribosomes and translation" or "transcription" or "protein fate" could apply to events in the nuc/cytosol, chloroplast, or mitochondria, which makes them quite ambiguous. Parsing out these terms for the different compartments would be much more informative.

iv) p. 7 line 42-51 refers to "two further cohorts of expressed genes representing organellar ribosomal protein genes… ." Where is this data shown? The only figure cited near this text is Fig. S2, but this does not illustrate the important points being made with these sentences.

v) Figures use sample numbers, but the narrative describing figures often refers instead to distance from the leaf base. Please help readers by using the same nomenclature in the narrative and figures.

vi) Fig S5a: I could not see the staining of plastid DNA referred to in the text.

vii) Fig 4j is described as showing expression of nuclear genes encoding chloroplast ribosomal proteins, but most of the genes shown do not encode chloroplast ribosomal proteins. SVR7 barely affects chloroplast translation, SCO1 is a translation elongation factor, and caution should be used for tRNA synthetases because many are dual targeted to mitochondria.

viii) Fig 4m. It would be very helpful to diagram the expression profiles of the major "PAPs" or "PTAC" proteins- proteins that tightly associate with the plastid-encoded RNA polymerase. These include PTAC2, PTAC10, MurE-like, PTAC12, FLN1, PTAC14 (see Pfalz and Pfannschmidt, TIPS 2013). It is of interest to know how their developmental profiles relate to those of sigma factors and the activation of PEP. It is also of interest to know whether the expression profile of PTAC12 stands out as distinct from the others, given the distinct functions attributed to its ortholog in Arabidopsis (Hemera).

ix) Figure 5a is labeled "photosynthesis", but the graph shows chlorophyll content.

x) p. 14, line 17 refers to genes for reaction centres in Fig 5c, but no true reaction center genes are shown.

xi) p. 15 refers to "previously identified chloroplast related transcriptional regulators". However, this section lumps together genes with very different types of function, including some whose functions are poorly understood. It is stated that it is "interesting" that GUN1 expression is maximally expressed at the transition stage. However, to evaluate this, it would be helpful to show the profile of chloroplast PPR proteins with well-defined functions in chloroplast biogenesis (eg expression of specific subunits of photosynthetic complexes - such as HCF152, PGR3, PPR10 - or expression of components of the translation machinery- such as PPR5 or PPR103).

xii) p. 16 line 5- and figure 6c needs more explanation. I could not follow the logic.

xiii) p. 17, line 10: the notion of distinct stages of chloroplast development has previously been put forward in other studies of monocot leaf development- such as the work from the Mullet lab (barley), work in maize from the Brutnell and van Wijk labs, and a maize chloroplast transcriptome/translatome paper from the Barkan lab.

**Reviewer 2**

**Are you able to assess all statistics in the manuscript, including the appropriateness of statistical tests used?**

Yes, and I have assessed the statistics in my report.

**Comments to author:**

The biogenesis of photosynthetically-active chloroplasts is an integral part of the cellular program of the host mesophyll cells. However, the cellular program controlling chloroplast biogenesis remains poorly understood. Taking advantage of the developmental gradient in the monocot wheat leaves, this comprehensive work dissects the distinct stages of chloroplast biogenesis in mesophyll-cell differentiation and establishes important connections linking cellular/plastidic morphology and division to transcriptomic changes of central components in cellular and hormonal signaling pathways. The manuscript is well written and the data beautifully presented. The great details of and novel insights into chloroplast biogenesis revealed by this study is truly impressive. I have only minor suggestions:

1. The authors have demonstrated the expression of distinct functional groups of genes in specific stages of chloroplast biogenesis. Because the basic/helix-loop-helix transcription factors Phytochrome-Interacting-Factors (PIFs) are a major nuclear hub repressing chloroplast biogenesis. I would suggest adding the expression profiles of the PIF family members in the analysis in Fig 6. One interesting question would be whether the PIFs play a role during the plastid phase to limit or delay the expression of photosynthesis-associated genes, and whether such repression is lifted in the chloroplast phase. With that said, because PIFs are involved in the regulation of chloroplast biogenesis in darkness and the authors looked at the proplastid-to-chloroplast transition in the light, it is also possible that PIFs are not at play in this process.

2. Page 8, Line 41: "In relation to chloroplast differentiation, this approach highlighted a possible role only for cytokinin, …" Please further elaborate on this point, because based on the indicated supplementary table and figure, it is not intuitive for the readers to arrive at this conclusion.

3. Page 15, Line 40/Fig6a: Because RCB is not included in Fig 6a, please also refer to the data in Fig 5e at the end of the sentence.

**Authors Response**

**Point-by-point responses to the reviewers’ comments:**

Reviewer comments:

Reviewer #1:

There is a wealth of information in this paper, and it will surely be of interest to the field. However, its impact would be greater if certain points were clarified and if prior work in this area were discussed in the context of the new data.

*Reply: We are very grateful for the very positive comments from the reviewer on the relevance and interest of our work. We also thank them for the constructive criticisms which have certainly helped improve the manuscript.*

>>i) Prior studies addressing related issues using cereal leaf blades are cited in the Introduction (references 7-14), but there is no attempt to integrate the findings from those studies with the results presented here. Furthermore, some seminal studies of this type are not cited at all. These include:

- classic work on chloroplast development along the barley leaf blade from the Mullet lab, which address things like the developmental profile of buildup of the chloroplast genome, translation machinery, etc.

- a comparative analysis of transcriptome dynamics along maize and rice seedling leaf blades (Brutnell lab).

It is important to discuss the body of prior work on this topic (both the cited papers, and those that should be cited) in the context of the new results here.

*Reply: We thank the reviewer for helping us better integrate our findings. The comparative analysis maize/rice was in fact already included in the introduction (now reference 13), and as it turns out we had also included one of the Mullet lab references in an earlier draft of the manuscript, which we had to trim in length and number of references before submission. We have included these two important references (6 and 7) as requested. Moreover, we have attempted to fully integrate previous observations with our data in the discussion, rewriting in particular paragraphs of the discussion section “Events in cellular and organellar life history” (pages 20-22).*

>>ii) It would be helpful to provide a brief explanation of (i) how the different "components" resulting from PCA are determined, what they signify and the interpretation of Figure 2a; (ii) the term "gene load factor"; (iii) what is shown on the X and Y axis in Fig S4.

*Reply: The examination of “load factors” in PCA is frequently used in multivariate analysis, and we have tried to exploit it in combination with the insights provided by GO term overrepresentation. The result of this combined strategy was revealing in our eyes. We have explained this in further detail and for a broader audience in the results, pages 6-7.*

>>iii) Fig. 2 and Fig S2. The functional terms "ribosomes and translation" or "transcription" or "protein fate" could apply to events in the nuc/cytosol, chloroplast, or mitochondria, which makes them quite ambiguous. Parsing out these terms for the different compartments would be much more informative.

*Reply: We thank the reviewer for this very useful suggestion. We had previously decided on a set of rules to identify the widest possible, yet confident, set of plastid-targeted proteins, described this in the methods and used it to select target genes for our gene network reconstruction (Fig. 7). We have now applied the same criteria to mitochondrial-targeted proteins. As a result of these two selections, a number of proteins appear as dual-targeted. We have now indicated this subcellular localisation (plastid, mitochondria, dual or neither) graphically in Fig. S2, and in the data for each individual gene in Table S2, for those three functional classes for which it is particularly relevant. We have also explained this in the results (pages 7-8).*

>>iv) p. 7 line 42-51 refers to "two further cohorts of expressed genes representing organellar ribosomal protein genes… ." Where is this data shown? The only figure cited near this text is Fig. S2, but this does not illustrate the important points being made with these sentences.

*Reply: This was in fact not directly shown, we referred to our interpretation of the fact that the heatmap of cytokinin signalling genes shows elevated expression at the time of fast greening (samples 6 to 13). We have indicated this in the text (page 9)*

>>v) Figures use sample numbers, but the narrative describing figures often refers instead to distance from the leaf base. Please help readers by using the same nomenclature in the narrative and figures.

*Reply: We had earlier referred to the distance from the leaf, as “sample 7” could potentially be interpreted as “half way in”, when it is physically much, much closer to the base than the tip. However, the reviewer is correct, the expression data are obviously obtained for “snapshot” samples. To help readers, we have carefully rewritten every such mention in the results, to include both the sample number and the physical position.*

>>vi) Fig S5a: I could not see the staining of plastid DNA referred to in the text.

*Reply: We have slightly adjusted the contrast of the images, and provided also an inset which enlarges a small area. Hopefully the reader will, using the images online, see this staining, even though it is obviously much, much less intense in plastids in meristematic than in chloroplasts of mature cells (as expected from the data in Fig. 4i).*

>>vii) Fig 4j is described as showing expression of nuclear genes encoding chloroplast ribosomal proteins, but most of the genes shown do not encode chloroplast ribosomal proteins. SVR7 barely affects chloroplast translation, SCO1 is a translation elongation factor, and caution should be used for tRNA synthetases because many are dual targeted to mitochondria.

*Reply: The reviewer is right, our intention was to display ribosome function-related processes. The legend (now for Fig. 4l) has now been replaced with “chloroplast translation”. We have retained the SVR7 gene, since the fact that its loss can suppress variegation shows that this loss slows down chloroplast growth in a significant manner. The tRNA synthetases, the reviewer is right, are dual-targeted, their expression profile telling us that the “bulk” of their expression is associated with the build-up of chloroplast, rather than mitochondrial, translation. However, to avoid ambiguities, we have replaced them with other chloroplast translation proteins with unique localisation to the chloroplast.*

>>viii) Fig 4m. It would be very helpful to diagram the expression profiles of the major "PAPs" or "PTAC" proteins- proteins that tightly associate with the plastid-encoded RNA polymerase. These include PTAC2, PTAC10, MurE-like, PTAC12, FLN1, PTAC14 (see Pfalz and Pfannschmidt, TIPS 2013). It is of interest to know how their developmental profiles relate to those of sigma factors and the activation of PEP. It is also of interest to know whether the expression profile of PTAC12 stands out as distinct from the others, given the distinct functions attributed to its ortholog in Arabidopsis (Hemera).

*Reply: Following the reviewer’s suggestion, we have displayed a number of nucleoid and pTAC proteins in Fig. 4j (also results page 13). They can be cross-checked with Fig. 4o, and this demonstrates that the PEP polymerase probably assembles with early sigma factors. The distinct profiles of pTAC12 and WHY3 become now very apparent. We believe our data provide a wealth of hypotheses for future examination.*

>>ix) Figure 5a is labeled "photosynthesis", but the graph shows chlorophyll content.

*Reply: This has been corrected.*

>>x) p. 14, line 17 refers to genes for reaction centres in Fig 5c, but no true reaction center genes are shown.

*Reply: We apologise. We should have said reaction centre-associated protein genes. This has been altered in the text (now page 15).*

>>xi) p. 15 refers to "previously identified chloroplast related transcriptional regulators". However, this section lumps together genes with very different types of function, including some whose functions are poorly understood. It is stated that it is "interesting" that GUN1 expression is maximally expressed at the transition stage. However, to evaluate this, it would be helpful to show the profile of chloroplast PPR proteins with well-defined functions in chloroplast biogenesis (eg expression of specific subunits of photosynthetic complexes - such as HCF152, PGR3, PPR10 - or expression of components of the translation machinery- such as PPR5 or PPR103).

*Reply: While the transcriptional regulators shown do include a range of functions, we believe it remains important to gage what power of regulation do they have, or how high in the candidate regulator list they should be. In mitochondrial biogenesis a protein which is not a transcription factor, but rather a transcription coactivator, PGC1alpha, has turned out to have a central role. The reviewer’s suggestion on GUN1, meanwhile, is very useful, and although GUN1 can be considered an indirect but important regulator of chloroplast biogenesis, we have removed it from Fig. 6 and incorporated it into a new Fig. S8 (additional description in the results on page 17) in which the expression of the genes the reviewer suggested is included. Interestingly, the expression of PGR3 was at odds with an exclusive role in RNA processing or translation for photosynthetic proteins, and indeed we found recent evidence in the literature (reference 48) of a dual role. This reassured us of the hypothesis-generating, validating or rejecting value of our data.*

>>xii) p. 16 line 5- and figure 6c needs more explanation. I could not follow the logic.

*Reply: We have incorporated a paragraph (page 18) describing the gene regulatory network reconstruction algorithm and our logic for employing and displaying it.*

>>xiii) p. 17, line 10: the notion of distinct stages of chloroplast development has previously been put forward in other studies of monocot leaf development- such as the work from the Mullet lab (barley), work in maize from the Brutnell and van Wijk labs, and a maize chloroplast transcriptome/translatome paper from the Barkan lab.

*Reply: We have been careful in our discussion to point this out, that our observations build upon that body of previous knowledge, but also to emphasise the clear distinction demonstrated by the quantitative nature of our analysis. Incidentally, since our previous submission, a very elegant study has been published (ref. 55) which also, literally, refers to “two distinct phases of chloroplast biogenesis”. However, we also note in our discussion that the phases we identify are not the same as those observed when cells with a substantial number of etioplasts, in a way semi-developed chloroplasts, are driven by light into full chloroplast development.*

Reviewer #2:

The manuscript is well written and the data beautifully presented. The great details of and novel insights into chloroplast biogenesis revealed by this study is truly impressive.

*Reply: We could have hardly hoped for more encouraging comments, and are truly grateful to the reviewer.*

1. The authors have demonstrated the expression of distinct functional groups of genes in specific stages of chloroplast biogenesis. Because the basic/helix-loop-helix transcription factors Phytochrome-Interacting-Factors (PIFs) are a major nuclear hub repressing chloroplast biogenesis. I would suggest adding the expression profiles of the PIF family members in the analysis in Fig 6. One interesting question would be whether the PIFs play a role during the plastid phase to limit or delay the expression of photosynthesis-associated genes, and whether such repression is lifted in the chloroplast phase. With that said, because PIFs are involved in the regulation of chloroplast biogenesis in darkness and the authors looked at the proplastid-to-chloroplast transition in the light, it is also possible that PIFs are not at play in this process.

*Reply: We thank the reviewer for this very useful suggestion. Following it, we decided to identify PIF homologues and display their expression, but to do so separately from that of candidate positive regulators. PIFs are now displayed in Fig. S9, described on pages 17-18. For completion, and while not direct transcriptional regulators, we have used the same rationale to display DET1 and COP1 expression. The known biology of PIFs in Arabidopsis shows them to be proteins accumulating in the dark and becoming labile in the light. Their expression in wheat (where, as in other monocots, homologues of Arabidopsis PIF1 and PIF3 are found) suggests more of a possible role arresting greening during the chloroplast growth stage if light becomes absent. However, we also used GENIE3 to assess the ranking of their regulatory power, and observed that one PIF3 homologue (PIF3.2) could indeed play a role during the plastid growth stage, presumably as a negative regulator as the reviewer suggested. This is presented in Fig. S9 and in the results (page 17) and discussion (Fig. 7 and page 21).*

2. Page 8, Line 41: "In relation to chloroplast differentiation, this approach highlighted a possible role only for cytokinin, …" Please further elaborate on this point, because based on the indicated supplementary table and figure, it is not intuitive for the readers to arrive at this conclusion.

*Reply: This was also raised by reviewer 1. We have addressed this in the text (page 9).*

3. Page 15, Line 40/Fig6a: Because RCB is not included in Fig 6a, please also refer to the data in Fig 5e at the end of the sentence.

*Reply: Thank you. Done (now page 17).*

*We have allowed ourselves to make a small addition which was not requested by the reviewers. It refers to the REC genes, with a role in determining the chloroplast coverage or cellular chloroplast index (calculated in Fig. 4g). We decided to display their expression in Fig. 4h, and this, interestingly, is consistent with primary roles for one of them in mitochondrial compartment, and for the others in chloroplast compartment determination. We would also like to note that it is our intention to discuss with Dr. Nicholas Provart the prospects of making our data available for display, mining and easy use by other investigators through the eFP tool at the Bioarray Resource (bar.utoronto.ca). We decided to not do so before final submission to avoid any further delays, but we believe this would make the data more widely accessible.*

*We hope the reviewers will find the changes have addressed all their requests, and that the manuscript will be accepted for publication in Genome Biology.*

**Second round of review**

**Reviewer 1**

This is a comprehensive and impressive analysis that is an invaluable resource for understanding leaf differentiation, chloroplast biogenesis, and the coordination of the two processes. The power of the leaf gradient system is abundantly clear, and I agree with these authors that this is a more biologically meaningful system than that used in the recent de-etiolation study. I am glad that the authors are working toward having their data displayed in an EF-P browser. If possible, please make it possible to query the data with gene IDs from orthologs/close homologs from Arabidopsis. Doing this will greatly increase the impact of this work.

The authors addressed most of my prior concerns and suggestions. However, I still have a few minor suggestions I hope they will consider.

1. Previously I requested more attention to integrating findings from prior studies that exploited the developmental gradient of the cereal leaf blade with this new data. Although prior publications are cited, those results are rarely explicitly mentioned in the context of the new data to highlight consistencies or discrepancies. For example, classic work on chloroplast development along the barley leaf blade from the Mullet lab addressed the buildup of chloroplast DNA content, translation machinery, etc. but aside from mentioning this work in a vague sense, those results are not connected to the similar results here. I could not find any comparison with the results from the cited comparative transcriptome along the maize and rice seedling leaf blade from the Brutnell lab. Similarly, a cited paper from the Barkan lab demonstrated a shift in the chloroplast transcriptome along the maize leaf blade (which reflects the NEP to PEP transition), and an increasing contribution of chloroplast translation to total cellular translation - results that tie in with findings in this work. When sigma factor expression dynamics are mentioned, it would be appropriate to discuss this in the context of the chloroplast genes each sigma is known to transcribe. A recent review article by Puthiyaveetil et al in BBA Gene Regulation summarizes the targets of the sigma factors in Arabidopsis and their expression patterns. It would be helpful to discuss the developmental dynamics of the sigmas in wheat in that context, even if very briefly.

2. Figure 2C and D. The functional terms listed in panel c do not always correspond with the bar graphs shown in panel d. For example, Module 1 in panel d lists cell cycle, transcription, ribosome&translation, whereas Module 1 in panel c does not list ribosome/translation but does list “cell growth”. Module 2 shows “hormones” and “transcription” to be similarly enriched in panel d, but only one of those is shown in panel c. It is important that the functional enrichment terms are the same in the two panels, and that all terms shown to be enriched in panel d are shown on the heat map in panel c.

3. Fig S2 and S3: I assume the numbered columns represent sample numbers, but this should be labeled on the plots (as in Figure 2c) for clarity.

4. p. 12 and Fig 4c show that FZL expression is not consistent with a role in plastid division. However, this gene was never claimed to function in plastid division. The original publication (which is cited as ref 32) and a more recent publication that is not cited (Liang et al, Plant Cell 2018) show that FZL functions in thylakoid biogenesis, which is consistent with its expression pattern. The discussion of these data should be corrected.

5. p. 13. When discussing cpDNA content along the leaf blade and its drop in the mature tissue, it would be relevant to point out that this differs from the conclusions made in maize from the Bendich group (JMB 2004, Planta 2006), but it is consistent with data in maize from the Barkan lab (Udy et al, Plant Phys 2012).

6. Fig 6c: each regulator seems to be associated with multiple columns. Please explain the distinction between the columns listed under each regulator. Sometimes, multiple regulators are grouped together at top with an underline. Please explain what this means.

7. p. 20, line 7: it was unclear which system was being referred to as being “more representative of the bulk of photosynthetic differentiation in nature”. I suspect the authors mean their system (and I would agree), but the sentence should be rephrased to make that clear.

**Authors Response**

**Point-by-point responses to the reviewers’ comments:**

Reviewer comments:

Reviewer #1:

This is a comprehensive and impressive analysis that is an invaluable resource for understanding leaf differentiation, chloroplast biogenesis, and the coordination of the two processes.

*Reply: We thank the reviewer once again.*

1. Previously I requested more attention to integrating findings from prior studies that exploited the developmental gradient of the cereal leaf blade with this new data. Although prior publications are cited, those results are rarely explicitly mentioned in the context of the new data to highlight consistencies or discrepancies. For example, classic work on chloroplast development along the barley leaf blade from the Mullet lab addressed the buildup of chloroplast DNA content, translation machinery, etc. but aside from mentioning this work in a vague sense, those results are not connected to the similar results here. I could not find any comparison with the results from the cited comparative transcriptome along the maize and rice seedling leaf blade from the Brutnell lab.

*Reply: We believe we had already done most of this, but perhaps in a way which was not sufficiently explicit. The key section of the discussion which attempted this was (page 20) that entitled “Events in cellular and organellar life history”. We wrote “It is possible, considering both published data and our analyses […] to recount…”. In the subsequent text we merged our observations with those in earlier, related analysis. For example, we wrote in the previously revised version:*

*“Once part of the elongating first leaf, MAJOR INVESTMENT CONTINUES ON TRANSCRIPTIONAL CONTROL PROCESSES AND PROTEIN SYNTHESIS [10, 11, 13] WHILE PROLIFERATION OF THOSE PROGENITOR CELLS [5, 6, 14] gradually ceases within 24h” (CAPS was text added in the first revision).*

*We have now re-revised this to highlight the earlier observations more clearly, as follows:*

*“major investment continues on transcriptional control processes and protein synthesis, AS LARGE-SCALE ANALYSES OBSERVED IN MAIZE AND RICE [10, 11, 13] while proliferation of those progenitor cells, PREVIOUSLY QUANTIFIED IN WHEAT AND BARLEY [5, 6, 14] gradually ceases within 24h”. The same approach has been taken in this section of the discussion, but also in other sections of both discussion and results.*

2. Similarly, a cited paper from the Barkan lab demonstrated a shift in the chloroplast transcriptome along the maize leaf blade (which reflects the NEP to PEP transition), and an increasing contribution of chloroplast translation to total cellular translation - results that tie in with findings in this work.

*Reply: We have made this explicit in the discussion (page 22) as follows: “consistent with observations in maize showing chloroplast translation becomes a more substantial component of total cellular translation capacity [9]”.*

3. When sigma factor expression dynamics are mentioned, it would be appropriate to discuss this in the context of the chloroplast genes each sigma is known to transcribe. A recent review article by Puthiyaveetil et al in BBA Gene Regulation summarizes the targets of the sigma factors in Arabidopsis and their expression patterns. It would be helpful to discuss the developmental dynamics of the sigmas in wheat in that context, even if very briefly.

*Reply: Transfer of functions between dicots and monocots is not always straight forward, but we thank the reviewer for asking us to delve further. We have interpreted the roles of “intermediate” and “late” sigma factors adding text, pages 22/23: “Interestingly, loss of the Arabidopsis homologues of only the intermediate wheat sigma factors, SIG2_2 and SIG6, peaking at the start of the chloroplast stage, results in reduced greening in Arabidopsis [59], while loss of other sigma factors can be compensated for”. And later on, page 23: “It is noteworthy that such late sigma factors are those which respond to blue light (SIG5) or redox signals (SIG1) modulating photosynthetic chloroplast composition in response to the environment [60]”. The Woodson and Puthiyaveethil references, 59 and 60, are also new.*

4. Figure 2C and D. The functional terms listed in panel c do not always correspond with the bar graphs shown in panel d. For example, Module 1 in panel d lists cell cycle, transcription, ribosome&translation, whereas Module 1 in panel c does not list ribosome/translation but does list “cell growth”. Module 2 shows “hormones” and “transcription” to be similarly enriched in panel d, but only one of those is shown in panel c. It is important that the functional enrichment terms are the same in the two panels.

*Reply: We have replaced “cell growth” with “cell growth / translation”, and “hormones” with “transcription and hormones” in Figure 2C. However, we noted in the figure legend that only a selection of terms was being used, and being expressed to highlight characteristic, or distinct, biological function. We have modified the figure legend as follows: “Some selected, overrepresented functional classification terms…”.*

5. Fig S2 and S3: I assume the numbered columns represent sample numbers.

*Reply: The label “samples” has been added.*

6. p. 12 and Fig 4c show that FZL expression is not consistent with a role in plastid division. However, this gene was never claimed to function in plastid division. The original publication (which is cited as ref 32) and a more recent publication that is not cited (Liang et al, Plant Cell 2018) show that FZL functions in thylakoid biogenesis, which is consistent with its expression pattern. The discussion of these data should be corrected.

*Reply: We did not say that FZL had been claimed to function in plastid division, but we did note that its mutant phenotype suggested such a role. We still want to highlight the surprising observation that no expression occurs at the time of plastid division. We do so with the following text (page 12): “The FZL gene has been shown to be involved in thylakoid biogenesis [31], but its mutant phenotype [32], with fewer, larger chloroplasts, is suggestive of a possible role in plastid proliferation; it is interesting to observe its later expression, between samples 6 and 12, 20 to 80 mm from the base (Fig. 4c), compatible with thylakoid development and incompatible with a role at the stage of division.”. We have also incorporated the second reference which the reviewer suggested use of (reference 31).*

7. p. 13. When discussing cpDNA content along the leaf blade and its drop in the mature tissue, it would be relevant to point out that this differs from the conclusions made in maize from the Bendich group (JMB 2004, Planta 2006), but it is consistent with data in maize from the Barkan lab (Udy et al, Plant Phys 2012).

*Reply: We have added the two references, within the following text (page 13): “The decrease, however, is consistent with a smaller decrease observed in mature maize leaf stages in one study [35], and does not support the near-total loss observed in another study [36]”.*

8. Fig 6c: each regulator seems to be associated with multiple columns. Please explain the distinction between the columns listed under each regulator. Sometimes, multiple regulators are grouped together at top with an underline. Please explain what this means.

*Reply: This has been clarified at the end of the legend of Fig. 6, as follows: “The separate columns represent the independently-computed values for the different homeologues (in most cases three) of each candidate regulator.”.*

9. p. 20, line 7: it was unclear which system was being referred to as being “more representative of the bulk of photosynthetic differentiation in nature”. I suspect the authors mean their system (and I would agree), but the sentence should be rephrased to make that clear.

*Reply: We have rephrased as follows: “with the processes we observed in the developing wheat leaf being more representative of the bulk of photosynthetic differentiation in nature”.*

*We believe we have now addressed these further requests, and hope that the manuscript will be accepted for publication in Genome Biology.*
